# Methicillin-Resistant *Staphylococcus aureus* ST80 Clone: A Systematic Review

**DOI:** 10.3390/toxins12020119

**Published:** 2020-02-14

**Authors:** Assia Mairi, Abdelaziz Touati, Jean-Philippe Lavigne

**Affiliations:** 1 Laboratoire d’Ecologie Microbienne, FSNV, Université de Bejaia, Bejaia 06000, Algeria; assia-mairi@hotmail.fr (A.M.); ziz1999@yahoo.fr (A.T.); 2 VBMI, INSERM U1047, Université de Montpellier, Service de Microbiologie et Hygiène Hospitalière, CHU Nîmes, 30029 Nîmes, France

**Keywords:** MRSA, ST80 clone, Panton–Valentine leukocidin, epidemic, worldwide diffusion, human, extra-human, decline

## Abstract

This review assessed the molecular characterization of the methicillin-resistant *Staphylococcus aureus* (MRSA)-ST80 clone with an emphasis on its proportion of total MRSA strains isolated, PVL production, *spa*-typing, antibiotic resistance, and virulence. A systematic review of the literature was conducted on MRSA-ST80 clone published between 1 January 2000 and 31 August 2019. Citations were chosen for a review of the full text if we found evidence that MRSA-ST80 clone was reported in the study. For each isolate, the country of isolation, the sampling period, the source of isolation (the type of infection, nasal swabs, or extra-human), the total number of MRSA strains isolated, number of MRSA-ST80 strains, antibiotic resistance patterns, PVL production, virulence genes, and *spa* type were recorded. The data from 103 articles were abstracted into an Excel database. Analysis of the data showed that the overall proportion of MRSA-ST80 has been decreasing in many countries in recent years. The majority of MRSA-ST80 were PVL positive with *spa*-type t044. Only six reports of MRSA-ST80 in extra-human niches were found. This review summarizes the rise of MRSA-ST80 and the evidence that suggests that it could be in decline in many countries.

## 1. Introduction

*Staphylococcus aureus* is an important bacterial pathogen frequently involved in hospital and community-acquired infections in humans. The most common sites affected are skin and soft tissue; infections in these sites include purulent (e.g., folliculitis, furuncles, carbuncles, impetigo, and mastitis) and toxinogenic infections (e.g., staphylococcal scalded skin syndrome and infections due to Panton–Valentine leukocidin (PVL)). Localized or systemic extensions of the infections are frequent, including bone and joint infections, bacteremia, pneumonia, and endocarditis. *S. aureus* has an extensive host range, including domestic cats and dogs, horses, goats, sheep, cattle, rabbits, pigs, and poultry [1,2].

Since the first isolation of methicillin-resistant *Staphylococcus aureus* (MRSA) in the early 1960s, MRSA strains have spread globally and caused countless nosocomial outbreaks, making MRSA one of the most common causes of nosocomial infections [3]. In addition to healthcare-associated MRSA (HA-MRSA), MRSA infections now also occur in the community (community-associated MRSA, CA-MRSA) [4]. The US Centers for Disease Control and Prevention (CDC) proposed a widely-adopted epidemiological definition of CA-MRSA as “any MRSA infection diagnosed in the outpatient setting or within 48 h of hospitalization, if the patient lacks the following HA-MRSA risk factors: hemodialysis, surgery, residence in a long-term care facility, or hospitalization during the previous year, the presence of an indwelling catheter or a percutaneous device at the time of culture.” All other MRSA infections are considered to be HA-MRSA. However, HA-MRSA could be considered to be community-associated if reported in hospital outside or within 48 h of admission [5].

*Staphylococcal cassette chromosome mec* (SCC*mec*) typing can be used to distinguish between CA-MRSA and HA-MRSA. CA-MRSA usually carries an SCC*mec* type IV or V [6]. However, isolates with a non-typeable SCC*mec* cassette would be missed, and SCC*mec*IV carrying HA-MRSA lineages, such as ST22-IV and ST5-IV, can be misclassified as CA-MRSA [7]. PVL has been proposed as a marker of CA-MRSA [8], however, some CA-MRSA PVL negative clones have also been reported [9,10].

The epidemiology of MRSA has changed since the late 1990s with the emergence of CA-MRSA. The CA-MRSA strains were detected worldwide, with different geographically distinct lineages, including the Southwest Pacific clone (ST30-IV) in East Asia and Oceania, USA400 (ST1-IV) and USA300 (ST8-IV) in the United States, and the European CA-MRSA clone (ST80-IV) in Europe, North Africa, and the Middle East.

## 2. Results

### 2.1. Clonal Complex 80 (CC80)

The first known isolate of the European CA-MRSA CC80 clone has been traced back to 1993 in Denmark before becoming established as the main CA-MRSA lineage throughout Europe [11]. It was assumed that this clone was imported into Europe by individuals having relations with the Middle East and Africa [12].

The phylogenetic analysis performed by Stegger et al. suggested that the MRSA CC80 lineage evolved from a PVL-positive MSSA ancestor that originated from sub-Saharan Africa. In addition, the authors suggested that the acquisition of both the SCC*mecA* element and the fusidic acid resistance determinant was done at the same moment. Furthermore, the diffusion of this clone in the early 1990s into Europe, the Middle East, and North Africa was probably associated with a mutation in the accessory regulatory gene *agrC* [10].

The typical molecular features of the European MRSA-ST80 clone isolates have been associated with SCC*mec* type IVc, ΦSa2 prophage harbored *lukS-PV* and *lukF-PV* genes (encoding the PVL), and an *agr* type III quorum-sensing system [13]. However, some sporadic reports of MRSA-ST80 did not meet these criteria. Thus, for example, an *agr* type I has been described in one isolate in Greece [14] and MRSA-ST80 strains harboring SCC*mec* type V and type I have been reported in Saudi Arabia [15,16] and Croatia, respectively [17]. Moreover, our analysis found that about 10% of MRSA-ST80 strains were PVL negative (Table S1).

Edslev et al. showed that a novel sublineage of CA-MRSA CC80 had been introduced into Denmark with different characteristics as compared with the typical European CA-MRSA clone (PVL negative, carriage of SCC*mec*-IVa element, and susceptibility to fusidic acid and aminoglycosides). These authors observed that all PVL-negative isolates in their study carried remnants of prophage ΦSa2, indicating that they were derived from a PVL-positive ancestor [18].

Diverse *spa* types (36 types) were identified among the MRSA-ST80 strains, with the dominance of types t044 (82.7%), t203 (6%), t131 (2.4%), t1028 (1.8%), t1200 (1.4%), and t376 (1%) (Table S1). This diversity of *spa* types confirms previous suggestions of the diverse route of acquisition of MRSA-ST80 [19,20].

### 2.2. Geographical Distribution of MRSA-ST80 in Humans

Since its first identification in Greece, in 1998 [21], the European clone MRSA-ST80 has been recognized as a cause of infections both in children and in adults in different parts of the world. However, there is a trend for a decrease in MRSA-ST80 prevalence observed in many countries.

Epidemiological data showed that MRSA-ST80 was spreading geographically in both hospital and community settings. It has been identified in at least 38 countries throughout Europe (23 countries), Middle East (10 countries), North Africa (3 countries), and Asia (2 countries).

In this section, we have used the term “prevalence” to express the proportion of MRSA-ST80 of total MRSA strains reported.

In an international study, among 3236 PVL+ MRSA isolates reported from 17 countries in the Americas, Europe, and Australia-Asia between 2009 and 2010, MRSA-ST80 was found to be exclusive to Europe. This study demonstrated that USA300 and related clones (ST8) have been progressively replacing the ST80 clone in several European countries [22]. However, El-Mahdy et al. examined 61 MRSA isolates from multinational patients at a hospital in Qatar from 2009 to 2010. The MRSA-ST80 strains were detected in 13.1% (8/61) of the total MRSA strains [23], suggesting the continuous dissemination of the clone, albeit at a reduced level.

#### 2.2.1. Asia

**Figure 1:**
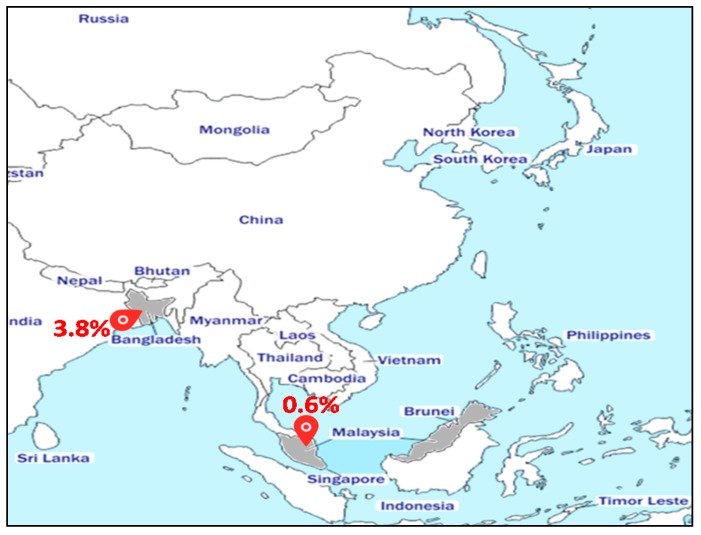
Prevalence of the methicillin-resistant *Staphylococcus aureus* (MRSA)-ST80 clone in Asia.

We identified two reports of MRSA-ST80 in Asian countries (Figure 1). In Malaysia, only one strain (1/154 MRSA) was identified from the nasal carriage of a dialyzed patient [24]. In Bangladesh, 3.8% (5/132) of MRSA strains have been assigned to ST80 [25]. Despite the low prevalence of MRSA-ST80 in Asia, most Asian hospitals are endemic for MRSA, and some Asian countries have among the highest MRSA prevalence in the world [26]. However, most available data are from high-income countries (e.g., Taiwan, Japan, Korea, Hong-Kong, and Singapore), with limited information from other nations [27]. Korea has a particularly high MRSA prevalence, with >70% among all clinical *S. aureus* isolates on the basis of regional surveillance data from 2011 [28]. These high MRSA rates are thought to be correlated to inappropriate therapy use, as well as high population density that facilitates rapid transmission of multidrug-resistant bacteria [28].

#### 2.2.2. North Africa

**Figure 2:**
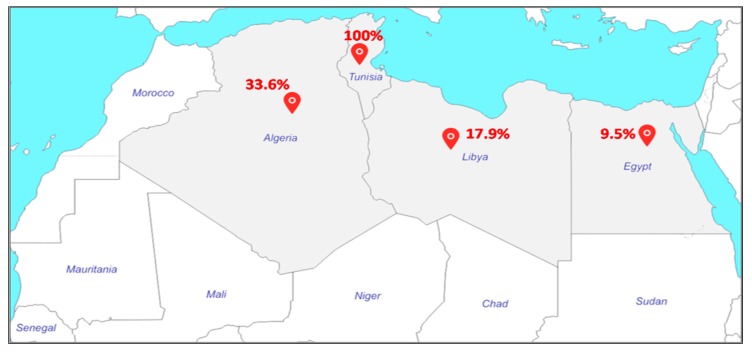
Prevalence of the MRSA-ST80 clone in North Africa.

MRSA-ST80 has been identified in Tunisia, Libya, and Algeria (Figure 2). In the two Tunisian studies, MRSA-ST80 was the only clone identified and was associated with infections in hospitalized children [29] and outpatients [30]. In Libya, Ahmed et al. reported that 17 of 95 MRSA strains (18%) were identified as MRSA-ST80 [31], whereas, in Algeria, different proportions of MRSA-ST80 have been reported. In Algiers, Antri et al. reported that the MRSA-ST80 was responsible for more than one-third of both community infections (35.7%) and hospital infections (35.8%) at the Mustapha Pacha hospital [32]. Djoudi et al. showed that 96% (24/25) of MRSA strains isolated from neonates and children at the Beni-Messous hospital (Algiers) were assigned to the ST80 clone [33]. In Eastern Algeria (Annaba), 21% (19/92) of MRSA strains isolated from skin and soft tissue infections (SSTIs) were characterized as ST80 clone [34]. In the same city, 13.7% (*n* = 10) of the 73 MRSA strains isolated from diabetic foot infections were assigned to ST80 clone [35]. In Western Algeria, Bekkhoucha et al. reported a proportion of 65% (15/23) of ST80 strains isolated from a hospital setting [36]. Finally, Djoudi et al. reported that 44.5% (4/9) of the total MRSA strains isolated from the nasal carriage of patients admitted to a general hospital and hemodialysis ward in Bejaia were identified as MRSA-ST80 [37].

MRSA-ST80 prevalence studies among clinical isolates in North Africa are numerous. However, most data published in North Africa on MRSA are from single-center studies, and information from regional surveillance systems is absent. In addition, most studies have relied on phenotypic methods to identify MRSA, and these tests could be less reliable than genotypic methods, especially as many studies did not integrate multilocus sequences typing (MLST) typing of MRSA strains.

#### 2.2.3. Middle East

**Figure 3:**
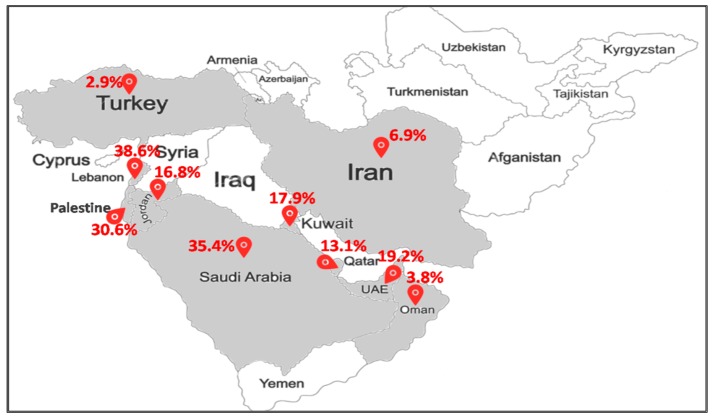
Prevalence of the MRSA-ST80 clone in the Middle East.

MRSA-ST80 has emerged as an important pathogen of community and hospital infections in many countries in the Middle East (Figure 3). Of the total MRSA strains, MRSA-ST80 prevalence was 3% (3/102) in Turkey [38], 4% (3/79) in Oman [39], 10% (2/21) in Egypt [40], 19% (10/52) in United Arab Emirates [41], and 31% (37/121) in Palestine [42]. In Kuwait, the reported proportions of MRSA-ST80 among MRSA strains were 38.4% (10/26) from 2001 to 2003 [43], 51% (69/135) from 2005 to 2006 [20], and 12% (3/25) from 2005 to 2007 [44]. MRSA-ST80 accounted for 7.5% (30/400) of all MRSA strains recovered from different clinical samples in 13 government Kuwait hospitals between 1992 and 2010 [19] and 7.8% (8/103) between 2006 and 2011 [45]. In Jordan, MRSA-ST80 strains were detected in 34.1% (14/41) of all MRSA strains recovered from infections from 2009 to 2010 [46]. Two studies reported that MRSA-ST80 strains represented 9.8% (4/41) and 5.4% (2/37) of all MRSA strains recovered from nasal and fecal carriage, respectively [47,48]. In two Iranian studies, MRSA-ST80 strains were found in 7.1% (5/70) of all MRSA strains recovered from patients from 2015 to 2017 [49] and 5.9% (1/17) in nasal carriage [50]. In Lebanon, MRSA-ST80 was recognized in 44% (41/93) from 2006 to 2007 [51] and 25.6% (10/39) in 2011 [52]. In Saudi Arabia, two studies reported nearly the same proportions of MRSA-ST80 in nasal carriage and infections in hospitals, that is, 35% (37/106) [16] and 36% (21/58) [53] of all MRSA strains.

The studies conducted in the Middle East have shown a spread of MRSA-ST80 in different countries within the region, with other clonal lineages, with country-based prevalence differences being detected from one country to another. We noted a high prevalence of MRSA-ST80 strains in Kuwait and Lebanon from 2005 to 2007 [20,51,54].

#### 2.2.4. Europe

In a multicenter study including 16 of the most populous countries in Europe, between 2000 and 2010, MRSA-ST80 accounted for 40.2% (51/127) of total MRSA strains isolated. These strains were recorded from Spain, France, Romania, Bulgaria, Poland, Czech Republic, Netherlands, Greece, Sweden, and Denmark [55].

**Figure 4:**
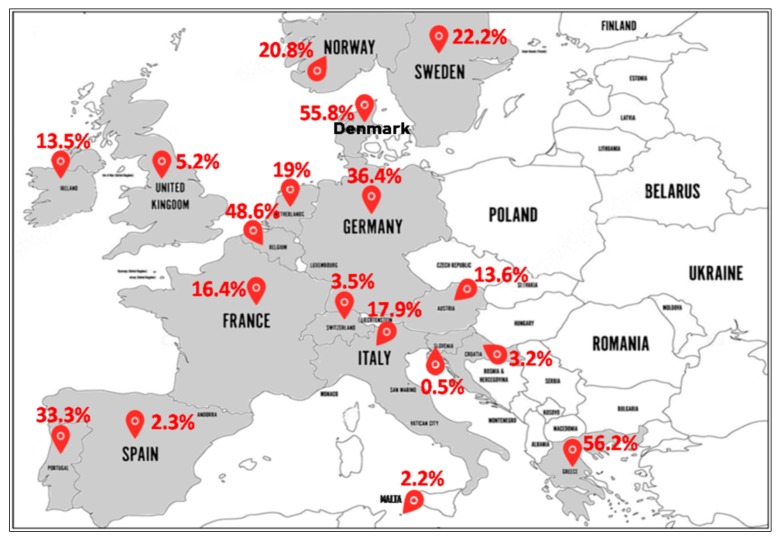
Prevalence of the MRSA-ST80 clone in Europe.

- North Europe (Figure 4): In Norway, MRSA-ST80 frequency was 24.5% (27/110) from 1991 to 2003 [56], 19.4% (13/67) from 1995 to 2003 [57], and 19% (34/179) in 2011 [58]. In Sweden, MRSA-ST80 strains were detected in 33.7% (35/104) [59] and 16.7% (36/216) [60] of all MRSA strains between 2000 and 2005. In Ireland, the reported percentages of MRSA-ST80 among MRSA strains were 8% (2/25) from 1999 to 2005 [61] and 14.2% (27/190) from 2002 to 2011 [62]. In England and Wales, Holmes et al. from 2002 to 2003 reported a low prevalence of PVL-positive *S. aureus* (1.6%) strains with different genetic backgrounds, although a large number belonged to the ST80 (80% of the PVL+ isolates) that was not geographically related [63]. The emergence of MRSA-ST80 was also noted in London in a collection of MRSA ciprofloxacin-resistant strains between 2000 and 2006 with a prevalence of 2.7% (12/450) [64]. Different percentages of MRSA-ST80 have been reported among MRSA strains in Denmark by many authors as follows: 100% (118/118) from 1997 to 2003 [65], 46.9% (38/81) in 2001 [11], 13.3% (19/143) from 2003 to 2004 [66] and 3.8% (13/341) from 2010 to 2013 [67]. MRSA-ST80 was recognized in 39.2% (206/526) among CA-MRSA strains from 1999 to 2006 [68]. McLaws et al. examined fusidic acid resistance in a collection of 1639 MRSA isolates collected in Denmark between 2003 and 2005 and found that MRSA-ST80 strains accounted for 61.2% (178/291) of the fusidic acid resistant isolates [69].
- South Europe (Figure 4): MRSA-ST80 was reported for the first time in Greece in 2003, which accounted for 9.3% (11/118) of all MRSA strains isolated [21]. Since then, several studies reporting different proportions of MRSA-ST80 among all MRSA isolates in Greece have been published including: 90.0% (18/20) in 2004 [70], 61.7% (428/694) from 2004 to 2005 [71], 100% (27/27) from 2006 to 2007 [72], 33.9% (61/180) from 2003 to 2009 [73], 13.7% (7/51) in 2009 [74], 61.5% (2838/4614) from 2001 to 2012 [75], 78.9% (45/57) from 2010 to. 2011 [76], and 4.8% (19/398) from 2000 to 2015 [77]. Doudoulakakis et al. reported a proportion of 95% (19/20) of selected MRSA isolates among Greek children recovering from pneumonia between 2007 and 2014 [14]. In Croatia, of the 248 MRSA isolates analyzed, 3.2% (8/248) were identified as MRSA-ST80 [17]. In Italy, frequencies of 10% (1/10) [78] and 22.2% (4/18) of MRSA-ST80 among CA-MRSA were reported [79]. In Slovenia, the proportion of MRSA-ST80 recorded was 0.5% (2/385) in a collection of CA-MRSA strains between 2006 and 2013 [80]. In Spain, frequencies of 1.9% (1/53) [81] and 2% (5/246) [82] of MRSA-ST80 among the MRSA PVL+ and CA-MRSA isolates were observed, respectively. Nearly the same percentage of 2.2% (1/45) of MRSA-ST80 among MRSA isolates was reported in Malta [83]. Of the three CA-MRSA strains isolated by Conceição et al., in Portugal, one of these strains was assigned to MRSA-ST80 clone [84].
- West Europe (Figure 4): In Austria, the percentages of MRSA-ST80 among the MRSA PVL+ strains analyzed were respectively 14.9% (14/94) from 2001 to 2006 [85] and 9.7% (3/31) from 2005 to 2010, respectively [86]. In Belgium, data from the reference laboratory for Staphylococci showed that MRSA-ST80 had been detected in 56.3% (9/16) of the MRSA PVL+ strains from 2002 to 2004 [87] and 47.8% (76/159) of the CA-MRSA PVL+ strains from 2005 to 2009 [88]. In Switzerland, the percentage of MRSA-ST80 among MRSA strains was 3.5% (7/200) from 2000 to 2005 [89]. Of the MRSA PVL+ strains described in the Netherlands, the frequency of MRSA-ST80 represented 60% (12/20) from 2000 to 2001 [90], rising to 79.6% (43/54) from 1998 to 2005 [91]. However, the proportion of the MRSA-ST80 clone was 1.1% (2/175) in a collection of MRSA collected from 2002 to 2006 [92] and 2.6% in a collection of 117 MRSA strains isolated from 2003 to 2010 [93]. Varying percentages of MRSA-ST80 were recorded in Germany as follows: 44.6% (33/74) of a low oxacillin-resistant isolates in 2005 [94], 68.4% (80/117) of the MRSA PVL+ isolates from 2005 to 2006 [95], 10.0% (8/80) from 2000 to 2007 [96], 0.3% (10/3207) of MRSA strains between two periods (1 February 2004 to 31 January 2005 and 1 February 2010 to 31 January 2011) [97], and 6.4% (6/94) of MRSA strains from 2012 to 2016 [98]. In a prospective multicenter study of MRSA isolates collected between September 2006 and February 2007 by 23 representative randomly selected French hospital laboratories, MRSA-ST80 strains were detected in 3.8% (4/105) of the MRSA strains isolated from invasive blood cultures [99]. In another French multicenter prospective study conducted in 2008, it was reported that among the 333 MRSA strains isolated, 91 (27.3%) were MRSA-ST80 [100]. Maugat et al. reported a low proportion of MRSA-ST80 of 0.4% (1/238) from 45 private-sector community-based medical laboratories in 2003 [101]. In a study conducted on *S. aureus* strains isolated from clinically relevant CA-SSTIs in 71 hospitals in November 2006, only one MRSA-ST80 strain (2.9%) was detected from the 34 CA-MRSA strains isolated [102].

All studies reporting MRSA-ST80 prevalence in European countries have shown the predominance of MRSA-ST80 clone. A significant proportion of MRSA-ST80 strains has been observed in Greece followed by Denmark, Belgium, and Germany. In these countries, a very low prevalence of MRSA strains has been recorded since the mid-1970s. However, since the late 1990s, an increase in new MRSA cases has been observed, especially involving community-associated infections. In addition, we noted high rates of MRSA-ST80 strains in these countries between 2004 and 2007, as in the Middle East.

#### 2.2.5. Other Parts of the World

If MRSA-ST80 strains have emerged and diffused in North Africa, the Middle East, and Europe, these isolates are absent in the other regions [22]. Only one case report has been published, that is, an imported strain, from Syria, has been described in USA, in 2015 [103]. Since this study, no other cases have been published, suggesting the absence of diffusion of this clone in this country. However, we could be ignoring that investigations of MRSA-ST80 are not fully performed in some parts of the world and its prevalence in these areas remains to be defined.

### 2.3. MRSA-ST80 in Extra-Human Niches

Six publications describing MRSA-ST80 in extra-human niches were found. In food items, one study reported the presence of three MRSA-ST80 PVL negative (*spa* type t1198) strains in ready-to-eat food in Singapore, in 2011 [104]. In Bangladesh, Islam et al. detected three MRSA-ST80 strains from retail foods, in which two were PVL positive (*spa* type t1198) and one PVL negative (*spa* type t8731) [105]. Concerning animals, Peeters et al. reported two strains of MRSA-ST80 PVL+ from nasal swabbing of pigs, in Belgium [106]. In Algeria, Agabou et al. reported two ST80 PVL+ isolates from camels and four isolates from sheep between 2015 and 2016 [107]. Mairi et al. studied a total of 2130 samples from different ecological niches (animal farms, companion animals, wild animals, food products, and aquatic environment) between January and July 2018. Seventeen MRSA-ST80 PVL+ strains were isolated from pets (*n* = 2), farm animals (*n* = 8), wild animals (*n* = 4), and food (*n* = 3) [108].

### 2.4. MRSA-ST80 Spread in the Community

Several risk factors for MRSA infection or colonization have been observed including previous hospitalization, admission to the ICU, residence in a nursing home, antibiotic therapy, foreign travel (North Africa, Middle East, Asia, and Mediterranean), and the presence of indwelling medical devices (e.g., surgery, catheter, prosthetic devices, and dialysis therapy). However, several publications have highlighted that MRSA-ST80 isolates could be recovered from patients without any risk factors for MRSA acquisition [11,84,103,109].

The ability of MRSA-ST80 to disseminate in the community has previously been documented. Transmission could occur by intensive contact sports (skin-to-skin, sharing equipment, and personal items) or via sports physicians and coaching staff [110]. Transmission of MRSA-ST80 among different host species in the context of households and veterinary practices has not been described, yet Drougka et al. demonstrated that dogs and cats can be “vectors” of MRSA-ST80 PVL+ strains causing severe infections in humans [111].

Urth et al. demonstrated that the MRSA-ST80 clone could spread in the general community in Denmark. They demonstrated that the clone was introduced from the Middle East on more than one occasion and that it was contagious within households and families, among children in kindergarten and school, and among adults at work. Moreover, the transmission was likely to have happened between an auxiliary nurse and the patient and could have occurred between patients and a practicing specialist [65].

Epidemiological data showed that MRSA-ST80 is widespread. Moreover, the dominance of MRSA-ST80, as the most common strain involved in SSTIs, and the overall increase in SSTIs suggested that there were specific properties of this clone to cause this kind of infections. Kolonitsiou et al. found that MRSA-ST80 PVL+ clone induces weak immune response of the host linked to a low level of production of pro-inflammatory cytokines by monocytes as compared with other lineages. This effect could favor the potential of the clone to evade host immune response and lead to persistent colonization. This could explain the ability of MRSA ST80 to disseminate successfully in the community and hospitals [112].

### 2.5. Antibiotic Susceptibility of MRSA-ST80

MRSA-ST80 isolates are of course resistant to all β-lactams. Concerning non-β-lactams antibiotics, MRSA-ST80 isolates differ from other CA-MRSA strains by often being resistant to fusidic acid, tetracycline, and kanamycin [80,113]. However, isolates susceptible to kanamycin, tetracycline, and fusidic acid that usually characterize MRSA-ST80 clone have been widely reported (Table S1). Thus, resistance rates of 91.6%, 86.5%, and 68.2% to kanamycin, fusidic acid, and tetracycline, respectively, were deduced from the analyzed articles. The resistance to these antibiotics is mediated by the *tetK* (tetracycline), *far1* (fusidic acid), and *aph(3′)-III* (kanamycin) genes (Table S1). In addition, several isolates expressed additional resistance to one or more antibiotics including erythromycin, clindamycin, ciprofloxacin, gentamicin, trimethoprim, mupirocin, and chloramphenicol. Classically, MRSA-ST80 isolates must be considered to be multidrug resistant bacteria because of resistance to more than three antibiotics families (Table S1).

### 2.6. Virulence of MRSA-ST80

Epidemiological studies of MRSA-ST80 PVL+ have revealed that these strains are primarily associated with recurrent, chronic, or particularly severe SSTIs, such as furuncles and abscesses [114]. This clone was also reported from other types of infections including fatal pneumonia and bloodstream infections [14,61,77,79]. In the study by Doudoulakakis et al., the authors reported that MRSA-ST80 PVL+ strains accounted for 95% of CA-MRSA pneumonia cases [14]. Nikolaras et al. showed that 8% of MRSA strains isolated from bloodstream infections were assigned to MRSA-ST80 clone [77]. In a collection of 94 MRSA-ST80 strains isolated in Lebanon and Jordan, 56% of these isolates were associated with SSTIs, 15% with respiratory tract infections, and 9% with bacteremia [115]. Djahmi et al. reported MRSA-ST80 PVL+ from inpatients with infected diabetic foot ulcers, requiring amputation (even if the link between these amputations and the presence of the clone was not determined) [35].

*S. aureus* expresses a large range of virulence factors among which secreted toxins play an important role in the ability to establish and maintain infection in humans. Among the toxins, the first group is constituted by hemolysins α, β, γ, δ, and the bicomponent leukotoxins (such as PVL). These toxins act on cell membranes forming lytic pores leading to the efflux of vital molecules, ions, and metabolites which destroy cells. The δ toxin is a sphingomyelinase that selectively activates and destroys human monocytes. A second group is composed of the superantigens (SAg) including TSST-1 and different enterotoxins. They act against hepatocytes by inhibiting the purifying activity of endotoxins, triggering more sensitivity to endotoxin shock. Moreover, this group of toxins involves a polyclonal activation of T lymphocytes, a massive release of cytokines, and the onset of toxic shock. A third group of toxins is formed by exfoliative toxins (EtA, EtB, and EtD) and serine proteases. They are responsible for the clinical manifestation of the staphylococcal scaled-skin syndrome (SSSS). These toxins create an intraepidermal cleavage by a proteolysis mechanism. Finally, the last group of toxins is called epidermal differentiation inhibitor (EDIN). These toxins target host Rho GTPases, favoring bacterial dissemination in tissues [116].

Among these virulence factors, the exfoliative toxin D (encoded by *etD* gene) has frequently been associated with MRSA-ST80 clone (Table S1). This toxin can contribute to a variety of infections by destroying epithelial barriers, helping bacteria to invade tissues for the exacerbation of infection. This can explain the success of the MRSA-ST80 clone, which carried the gene *etD* in combination with the *pvl* genes [63]. The *edinB* gene, which is on the same operon as *etD*, was also frequently observed in MRSA-ST80 strains (Table S1). Courjon et al. demonstrated that the chromosomally encoded EdinB in MRSA-ST80 contributes to the onset of bacteremia during the course of pneumonia [117]. Microarray analysis performed on MRSA-ST80 strains revealed these isolates harbored several types of virulence factors, in addition to *pvl*, *etD* and *edinB* (Table S1).

Studies reporting the MRSA-ST80 virulence genes in non–human sources are rare. Aung et al. reported MRSA-ST80 strains in retail food, detected enterotoxin encoding genes (*seb*, *sek*) and exfoliative-encoding gene (*etd*). No other toxin genes were detected [104]. Agabou et al. reported colonization of livestock animals (camels and sheep) with MRSA-ST80 in Algeria which harbored hemolysins (*hla, hld*, and *hlgA*), numerous microbial surface components recognizing adhesive matrix molecules (MSCRAMMs) (*bbp, ebpS, clfA, clfB, fib, fnbA,* and *fnbB*), and biofilm production (*icaA, icaC,* and *icaD*) genes [107]. Mairi et al. reported 20 MRSA-ST80 isolates in different niches [108], identifying the same genes as previously noted by Agabou et al. [107]. Finally, Peeters et al. reported two MRSA-ST80 isolates from nasal swabs of pig farms that carried the following genes: *fnbB, edinB, sasG, sak, scn, hla, hld, hIII, lukDE/F/S, seg, sei, selm, selo, selu, etD,* and *cap8* [106].

The management of infections due to the MRSA-ST80 clone follows recommendations mainly based on expert opinions [118]. The main points are the following: (i) the abscesses must be irrigated and drained; (ii) antibiotic choices must promote parenteral or oral antibiotics with an anti-toxin effect (e.g., linezolide, clindamycin, rifampicin); and (iii) the adjunction of intravenous immunoglobulin could be needed in severe cases to neutralize the exotoxins and superantigens. In association, the measures and procedures of infection prevention and control must be applied with the use of standard (hand hygiene) contact and respiratory precautions [119]. This includes isolation in a single room, use of personal protective equipment, meticulous hand hygiene, and environmental cleaning.

### 2.7. MRSA Typing Methods and Techniques

Molecular typing methods constitute invaluable tools for tracing the spread of particular strains, discovering the route of dissemination and the potential reservoirs. Furthermore, molecular typing contributes to the comprehensive understanding of the epidemiology of infection and facilitates infection control measures, as well as management [120]. From the analyzed articles included in this review, several methods were used for MRSA typing.

The *SCCmec*-typing method is a PCR-based method and detects the SCC*mec* cassette type but not its structure, and therefore is less discriminative [121]. This characterization method has become an important technique for distinguishing between HA-MRSA and CA-MRSA [122]. However, this technique cannot be applied to the detection of novel SCC*mec* types [123].

*Staphylococcal* protein A (*spa*) typing is a sequence-based tool based on the sequencing of the highly variable X fragment of the *spa* gene region encoding protein A. In addition to its simplicity and relative low cost, the integration of the *spa* types into an international database and its unified nomenclature provides another important advantage. The use of these typing tools contributes to understanding clonal diversity and transmission of MRSA in the hospital and community settings [124]. Infrequently, when misclassification or non-typeability is a concern, sophisticated typing tools, such as PFGE and MLST, are informative supplementary additions [125,126].

The multilocus sequences typing (MLST) approach was established to analyze the allelic sequence of short internal DNA fragments (450 to 500 bp) of seven constitutively *S. aureus* housekeeping genes [127]. This method is an interesting tool for screening and studying the molecular evolution of MRSA and the results are comparable between laboratories around the world via a web-based database [128]. However, the major drawback to MLST is its cost, and therefore it is not suitable for diagnostic purposes [129].

The pulsed-field gel electrophoresis (PFGE) approach was once considered the gold standard for MRSA typing in outbreak investigations and has been widely used for the understanding of the epidemiology of both endemic and epidemic MRSA strains [9]. This method is extremely helpful in short-term investigations and successful at the national levels in some countries but not internationally [130].

A DNA microarray analysis, also known as biochip or DNA chip analysis, is a very useful technique employed for MRSA typing which uses a collection of DNA probes attached to a solid surface in an ordered manner. It is an efficient tool for the detection of antibiotic resistance and virulence genes simultaneously, and therefore has the potential to detect new epidemiological markers for clones [131]. Whereas DNA microarray analysis is adequate, discriminatory power, high reproducibility, and low cost within and between different laboratories needs to be established. The limitation to this technique is that the detection is limited only to sequences that are included in the array [95,132].

Whole genome sequencing (WGS) is considered to be a very attractive tool for epidemiological purposes, and, in the near future, it is expected that this technology could take over from routine tools for the identification and characterization of MRSA strains. Currently, this approach permits the genetic relatedness between bacteria and manages the outbreak investigations based on sequence analysis of the whole genome [133,134]. Subsequently, the WGS determines the virulence genes contained on the MRSA strains, as well as locally and globally determines the emerging infectious strains. However, the most important challenge for this technology is the interpretation and computation of the huge set of data. In addition, WGS requires significant computer resources and well-trained bioinformaticians [135].

## 3. Discussion

MRSA-ST80 infection is still a major public health problem in many countries in different parts the world. Some signs indicate that MRSA-ST80 could have a declining prevalence in several countries, especially in Europe and the Middle East. It is likely that its decline is due to the introduction of new epidemic clones (clonal replacement) which have succeeded in several geographical areas.

While MRSA-ST80 is still a major problem in many countries, there are hints that it could be decreasing in prevalence or, at least, not continuing its expansion. The majority of studies reporting MRSA-ST80 clone were conducted during the period from 2000 to 2013. As of mid-2015, few articles reporting MRSA have been published. Over the last three years, to the best of our knowledge, only three studies reporting MRSA-ST80 clone have been published. These three articles were published only from North Africa [107,108] and Iran [50]. In general, MRSA infections are declining in certain geographic areas, especially in hospital-associated infections [136,137]. MRSA has decreased in European countries, Australia, Taiwan, and South Africa. However, data from the rest of Africa, Greece, and many parts of Asia suggest stable or increasing MRSA prevalence [138]. The reasons for this MRSA-specific decline are not fully understood. It is possible that the MRSA decreased due to control measures which included bundled measures such as identification and isolation of MRSA carriers, hand hygiene, and antibiotic policy [139].

Epidemiological studies using molecular typing methods have indicated the dissemination of a few highly epidemic clones worldwide. The introduced epidemic clones belong to a predominant sequence type ST239 (SCC*mec*III/IIIA-Brazilian) and ST22 (SCC*mec*IV-EMRSA-15) followed by ST5 (SCC*mec*II-New York/Japan, and SCC*mec*IV-Pediatric), ST45 (SCC*mec*IV-Berlin), and ST247 (SCC*mec*IA-Iberian).

In addition to changes in MRSA infection rates, the molecular epidemiology of these organisms has undergone alterations over time. Several MRSA lineages have been successful in a particular geographic area, have peaked and, then, declined before disappearing with the emergence of other epidemic clones. This phenomenon of clonal replacement has been observed worldwide and the reasons for the decline of some lineages are mysterious. For example, the Hungarian clone (MRSA-ST239-III) was the most predominant clone between 1994 and 1998 in Hungary [140,141]. This clone disappeared and was replaced by the southern German clone (MRSA-ST228-I) and the New York/Japan epidemic clone (MRSA-ST5-II) between 2001 and 2004 [142]. Recent studies reported the local predominance of this clone in many countries including European countries [143,144], the Middle East, Asia, and South America [145]. Another epidemic clone, MRSA-ST22, has been predominately identified in recent years throughout the Middle East [44,45,146,147,148,149,150,151,152,153,154], Europe [155,156,157,158,159,160], and Asia [161,162,163]. For North Africa, the lower number of studies on MRSA means that MRSA lineages remained unclear.

Efforts to understand the main factors contributing to the reduced prevalence of MRSA ST80, as well as host and environmental factors associated with its decline, could help us manage future outbreaks of MRSA. It would be interesting to consider whole genome analysis in future studies, which could give a particular vision of the expansion of this clone and its decline. In addition, surveillance programs to detect new and expanding MRSA clones would help us better understand how lineages develop to occupy different niches.

## 4. Materials and Methods

### 4.1. Literature Review

We systematically reviewed the literature to identify all peer-reviewed publications, including genotyping information on MRSA isolates.

A PubMed search was conducted for citations related to MRSA ST80 clone published from 2003 to 2019 using the following search criteria: (“2003/01/01” (date—publication): “2019/08/31” (date—publication)) and “methicillin-resistant *Staphylococcus aureus* ST80”, “oxacillin-resistant *Staphylococcus aureus* ST80”, “MRSA ST80”, “ORSA ST80”, “methicillin-resistant *Staphylococcus aureus* CC80”, “oxacillin-resistant *Staphylococcus aureus* CC80”, “MRSA CC80”, and “ORSA CC80”. Citations were chosen for a review of the full text if ST80 clone was reported in the study or genotyping of MLST was performed for the study. Studies were then sorted by the country of sampling.

### 4.2. Data Abstraction

The search criteria identified isolates from 103 articles for inclusion. Data on 18,510 isolates (5571 MRSA-ST80/18, 510 MRSA) were, then, abstracted into an Excel database. For each isolate, the country of collection, dates of isolation, source of the isolation (animal species, human or other niches), types of specimens (infection or carriage), number of MRSA strains, number of MRSA ST80, and antibiotic resistance pattern were recorded.

For each isolate, the following genotyping information was recorded if it was provided in the article: SCC*mec* type; *spa* type; *agr* type; the presence or absence of PVL; virulence genes, and antibiotic resistance genes (Table S1).

## Supplementary Materials

The following are available online at https://www.mdpi.com/2072-6651/12/2/119/s1, Table S1: Characteristics of MRSA-ST80 isolates published and studied in this review.

## References

[1] Peacock S.J., Paterson G.K. (2015). Mechanisms of methicillin resistance in *Staphylococcus aureus*. Annu. Rev. Biochem..

[2] Otto M. (2012). MRSA virulence and spread. Cell Microbiol..

[3] Harkins C.P., Pichon B., Doumith M. (2017). Methicillin-resistant *Staphylococcus aureus* emerged long before the introduction of methicillin into clinical practice. Genome Biol..

[4] Dastgheyb S.S., Otto M. (2015). Staphylococcal adaptation to diverse physiologic niches: An overview of transcriptomic and phenotypic changes in different biological environments. Future Microbiol..

[5] Khan A., Wilson B., Gould I.M. (2018). Current and future treatment options for community-associated MRSA infection. Expert Opin. Pharmacother..

[6] Asghar A.H. (2014). Molecular characterization of methicillin-resistant *Staphylococcus aureus* isolated from tertiary care hospitals. Pak. J. Med. Sci..

[7] Otter J.A., French G.L. (2012). Community-associated meticillin-resistant *Staphylococcus aureus*: The case for a genotypic definition. J. Hospit. Infect..

[8] Valle D.L., Paclibare P.A.P., Cabrera E.C., Rivera W.L. (2016). Molecular and phenotypic characterization of methicillin-resistant *Staphylococcus aureus* isolates from a tertiary hospital in the Philippines. Trop. Med. Health.

[9] Lakhundi S., Zhang K. (2018). Methicillin-resistant *Staphylococcus aureus*: Molecular characterization, evolution, and epidemiology. Clin. Microbiol. Rev..

[10] Stegger M., Wirth T., Andersen P.S., Skov R.L., De Grassi A., Simões P.M., Tristan A., Petersen A., Aziz M., Kiil K. (2014). Origin and evolution of european community-acquired methicillin-resistant *Staphylococcus aureus*. mBio.

[11] Faria N.A., Oliveira D.C., Westh H., Monnet D.L., Larsen A.R., Skov R., de Lencastre H. (2005). Epidemiology of emerging methicillin-resistant *Staphylococcus aureus* (MRSA) in Denmark: A nationwide study in a country with low prevalence of MRSA infection. J. Clin. Microbiol..

[12] Larsson A.-K., Gustafsson E., Johansson P.J.H., Odenholt I., Petersson A.C., Melander E. (2014). Epidemiology of MRSA in southern Sweden: Strong relation to foreign country of origin, health care abroad and foreign travel. Eur. J. Clin. Microbiol. Infect. Dis..

[13] Monecke S., Ehricht R., Slickers P., Wiese N., Jonas D. (2009). Intra-strain variability of methicillin-resistant *Staphylococcus aureus* strains ST228-MRSA-I and ST5-MRSA-II. Eur. J. Clin. Microbiol. Infect. Dis..

[14] Doudoulakakis A.G., Bouras D., Drougka E., Kazantzi M., Michos A., Charisiadou A., Spiliopoulou I., Lebessi E., Tsolia M. (2016). Community-associated *Staphylococcus aureus* pneumonia among Greek children: Epidemiology, molecular characteristics, treatment, and outcome. Eur. J. Clin. Microbiol. Infect. Dis..

[15] Abou Shady H.M., Bakr A.E.A., Hashad M.E., Alzohairy M.A. (2015). *Staphylococcus aureus* nasal carriage among outpatients attending primary health care centers: A comparative study of two cities in Saudi Arabia and Egypt. Braz. J. Infect. Dis..

[16] Alkharsah K.R., Rehman S., Alkhamis F., Alnimr A., Diab A., Al-Ali A.K. (2018). Comparative and molecular analysis of MRSA isolates from infection sites and carrier colonization sites. Ann. Clin. Microbiol. Antimicrob..

[17] Budimir A., Deurenberg R.H., Bosnjak Z., Stobberingh E.E., Cetkovic H., Kalenic S. (2010). A variant of the Southern German clone of methicillin-resistant *Staphylococcus aureus* is predominant in Croatia. Clin. Microbiol. Infect..

[18] Edslev S.M., Westh H., Andersen P.S., Skov R., Kobayashi N., Bartels M.D., Vandenesch F., Petersen A., Worning P., Larsen A.R. (2018). Identification of a PVL-negative SCC*mec*-IVa sublineage of the methicillin-resistant *Staphylococcus aureus* CC80 lineage: Understanding the clonal origin of CA-MRSA. Clin. Microbiol. Infect..

[19] Boswihi S.S., Udo E.E., Al-Sweih N. (2016). Shifts in the clonal distribution of methicillin-resistant *Staphylococcus aureus* in Kuwait hospitals: 1992–2010. PLoS ONE.

[20] Udo E.E., Sarkhoo E. (2010). The dissemination of ST80-SCC*mec*-IV community-associated methicillin resistant *Staphylococcus aureus* clone in Kuwait hospitals. Ann. Clin. Microbiol. Antimicrob..

[21] Aires de Sousa M., Bartzavali C., Spiliopoulou I., Sanches I.S., Crisóstomo M.I., de Lencastre H. (2003). Two international methicillin-resistant *Staphylococcus aureus* clones endemic in a university hospital in Patras, Greece. J. Clin. Microbiol..

[22] Macedo-Viñas M., Conly J., Francois P., Aschbacher R., Blanc D.S., Coombs G., Daikos G., Dhawan B., Empel J., Etienne J. (2014). Antibiotic susceptibility and molecular epidemiology of Panton-Valentine leukocidin-positive meticillin-resistant *Staphylococcus aureus*: An international survey. J. Glob. Antimicrob. Resist..

[23] El-Mahdy T.S., El-Ahmady M., Goering R.V. (2014). Molecular characterization of methicillin-resistant *Staphylococcus aureus* isolated over a 2-year period in a Qatari hospital from multinational patients. Clin. Microbiol. Infect..

[24] Ahmad N., Ruzan I.N., Abd Ghani M.K., Hussin A., Nawi S., Aziz M.N., Maning N., Eow V.L.K. (2009). Characteristics of community- and hospital-acquired meticillin-resistant *Staphylococcus aureus* strains carrying SCC*mec* type IV isolated in Malaysia. J. Med. Microbiol..

[25] Haque N., Aung M.S., Paul S.K., Bari M.S., Ahmed S., Sarkar S.R., Roy S., Nasreen S.A., Mahmud M.C., Hossain M.A. (2019). Molecular epidemiological characterization of methicillin-susceptible and -resistant *Staphylococcus aureus* isolated from skin and soft tissue infections in Bangladesh. Microb. Drug Resist..

[26] Latha T., Anil B., Manjunatha H., Chiranjay M., Elsa D., Baby N., Anice G. (2019). MRSA: The leading pathogen of orthopedic infection in a tertiary care hospital, South India. Afr. Health Sci..

[27] Mendes R.E., Mendoza M., Banga Singh K.K., Castanheira M., Bell J.M., Turnidge J.D., Lin S.S.F., Jones R.N. (2013). Regional resistance surveillance program results for 12 Asia-Pacific nations (2011). Antimicrob. Agents Chemother..

[28] Chen C.-J., Huang Y.-C. (2014). New epidemiology of *Staphylococcus aureus* infection in Asia. Clin. Microbiol. Infect..

[29] Ben N.M., Merghni A., Mastouri M. (2014). Genotyping of Methicillin Resistant *Staphylococcus aureus* Strains Isolated from Hospitalized Children. Int. J. Pediatr..

[30] Ben Nejma M., Mastouri M., Bel Hadj Jrad B., Nour M. (2013). Characterization of ST80 Panton-Valentine leukocidin-positive community-acquired methicillin-resistant *Staphylococcus aureus* clone in Tunisia. Diagn. Microbiol. Infect. Dis..

[31] Ahmed M.O., Baptiste K.E., Daw M.A., Elramalli A.K., Abouzeed Y.M., Petersen A. (2017). Spa typing and identification of *pvl* genes of meticillin-resistant *Staphylococcus aureus* isolated from a Libyan hospital in Tripoli. J. Glob. Antimicrob. Resist..

[32] Antri K., Rouzic N., Dauwalder O., Boubekri I., Bes M., Lina G., Vandenesch F., Tazir M., Ramdani-Bouguessa N., Etienne J. (2011). High prevalence of methicillin-resistant *Staphylococcus aureus* clone ST80-IV in hospital and community settings in Algiers. Clin. Microbiol. Infect..

[33] Djoudi F., Bonura C., Benallaoua S., Touati A., Touati D., Aleo A., Cala C., Fasciana T., Mammina C. (2013). Panton-Valentine leukocidin positive sequence type 80 methicillin-resistant *Staphylococcus aureus* carrying a *Staphylococcal* cassette chromosome *mec* type IVc is dominant in neonates and children in an Algiers hospital. New Microbiol..

[34] Alioua M.A., Labid A., Amoura K., Bertine M., Gacemi-Kirane D., Dekhil M. (2014). Emergence of the European ST80 clone of community-associated methicillin-resistant *Staphylococcus aureus* as a cause of healthcare-associated infections in Eastern Algeria. Med. Mal. Infect..

[35] Djahmi N., Messad N., Nedjai S., Moussaoui A., Mazouz D., Richard J.-L., Sotto A., Lavigne J.-P. (2013). Molecular epidemiology of *Staphylococcus aureus* strains isolated from inpatients with infected diabetic foot ulcers in an Algerian University Hospital. Clin. Microbiol. Infect..

[36] Bekkhoucha S.N., Cady A., Gautier P., Itim F., Donnio P.-Y. (2009). A portrait of *Staphylococcus aureus* from the other side of the Mediterranean Sea: Molecular characteristics of isolates from Western Algeria. Eur. J. Clin. Microbiol. Infect. Dis..

[37] Djoudi F., Benallaoua S., Aleo A., Touati A., Challal M., Bonura C., Mammina C. (2015). Descriptive epidemiology of nasal carriage of *Staphylococcus aureus* and methicillin-resistant *Staphylococcus aureus* among patients admitted to two healthcare facilities in Algeria. Microb. Drug Resist..

[38] Oksuz L., Dupieux C., Tristan A., Bes M., Etienne J., Gurler N. (2013). The high diversity of MRSA clones detected in a university hospital in istanbul. Int. J. Med. Sci..

[39] Udo E.E., Al-Lawati B.A.-H., Al-Muharmi Z., Thukral S.S. (2014). Genotyping of methicillin-resistant *Staphylococcus aureus* in the Sultan Qaboos University Hospital, Oman reveals the dominance of Panton–Valentine leucocidin-negative ST6-IV/t304 clone. New Microbes New Infect..

[40] Enany S., Yaoita E., Yoshida Y., Enany M., Yamamoto T. (2010). Molecular characterization of Panton-Valentine leukocidin-positive community-acquired methicillin-resistant *Staphylococcus aureus* isolates in Egypt. Microbiol. Res..

[41] Sonnevend Á., Blair I., Alkaabi M., Jumaa P., Al Haj M., Ghazawi A., Akawi N., Jouhar F.S., Hamadeh M.B., Pál T. (2012). Change in meticillin-resistant *Staphylococcus aureus* clones at a tertiary care hospital in the United Arab Emirates over a 5-year period. J. Clin. Pathol..

[42] Laham N.A., Mediavilla J.R., Chen L., Abdelateef N., Elamreen F.A., Ginocchio C.C., Pierard D., Becker K., Kreiswirth B.N. (2015). MRSA clonal complex 22 strains harboring toxic shock syndrome toxin (TSST-1) are endemic in the primary hospital in Gaza, Palestine. PLoS ONE.

[43] Udo E.E., O’Brien F.G., Al-Sweih N., Noronha B., Matthew B., Grubb W.B. (2008). Genetic lineages of community-associated methicillin-resistant *Staphylococcus aureus* in Kuwait hospitals. J. Clin. Microbiol..

[44] Alfouzan W., Dhar R., Udo E. (2017). Genetic lineages of methicillin-resistant *Staphylococcus aureus* acquired during admission to an intensive care unit of a general hospital. Med. Princ. Pract..

[45] Udo E.E., Al-Sweih N. (2017). Dominance of community-associated methicillin-resistant *Staphylococcus aureus* clones in a maternity hospital. PLoS ONE.

[46] Bazzoun D.A., Harastani H.H., Shehabi A.A., Tokajian S.T. (2014). Molecular typing of *Staphylococcus aureus* collected from a major hospital in Amman, Jordan. J. Infect. Dev. Ctries.

[47] Al-Bakri A.G., Al-Hadithi H., Kasabri V., Othman G., Kriegeskorte A., Becker K. (2013). The epidemiology and molecular characterization of methicillin-resistant *Staphylococci* sampled from a healthy Jordanian population. Epidemiol. Infect..

[48] Khalil W., Hashwa F., Shihabi A., Tokajian S. (2012). Methicillin-resistant *Staphylococcus aureus* ST80-IV clone in children from Jordan. Diagn. Microbiol. Infect. Dis..

[49] Ohadian Moghadam S., Modoodi Yaghooti M., Pourramezan N., Pourmand M.R. (2017). Molecular characterization and antimicrobial susceptibility of the CA-MRSA isolated from healthcare workers, Tehran, Iran. Microb. Pathog..

[50] Goudarzi M., Navidinia M., Beiranvand E., Goudarzi H. (2018). Phenotypic and molecular characterization of methicillin-resistant *Staphylococcus aureus* clones carrying the Panton-Valentine leukocidin genes disseminating in Iranian hospitals. Microb. Drug Resist..

[51] Tokajian S.T., Khalil P.A., Jabbour D., Rizk M., Farah M.J., Hashwa F.A., Araj G.F. (2010). Molecular characterization of *Staphylococcus aureus* in Lebanon. Epidemiol. Infect..

[52] Harastani H.H., Araj G.F., Tokajian S.T. (2014). Molecular characteristics of *Staphylococcus aureus* isolated from a major hospital in Lebanon. Int. J. Infect. Dis..

[53] Monecke S., Skakni L., Hasan R., Ruppelt A., Ghazal S.S., Hakawi A., Slickers P., Ehricht R. (2012). Characterisation of MRSA strains isolated from patients in a hospital in Riyadh, Kingdom of Saudi Arabia. BMC Microbiol..

[54] Udo E.E., Sarkhoo E. (2010). Genetic analysis of high-level mupirocin resistance in the ST80 clone of community-associated meticillin-resistant *Staphylococcus aureus*. J. Med. Microbiol..

[55] Rolo J., Miragaia M., Turlej-Rogacka A., Empel J., Bouchami O., Faria N.A., Tavares A., Hryniewicz W., Fluit A.C., de Lencastre H. (2012). High genetic diversity among community-associated *Staphylococcus aureus* in Europe: Results from a multicenter study. PLoS ONE.

[56] Fossum A.E., Bukholm G. (2006). Increased incidence of methicillin-resistant *Staphylococcus aureus* ST80, novel ST125 and SCC*mec*IV in the south-eastern part of Norway during a 12-year period. Clin. Microbiol. Infect..

[57] Hanssen A.-M., Fossum A., Mikalsen J., Halvorsen D.S., Bukholm G., Sollid J.U.E. (2005). Dissemination of community-acquired methicillin-resistant *Staphylococcus aureus* clones in northern Norway: Sequence types 8 and 80 predominate. J. Clin. Microbiol..

[58] Monecke S., Aamot H.V., Stieber B., Ruppelt A., Ehricht R. (2014). Characterization of PVL-positive MRSA from Norway. APMIS.

[59] Fang H., Hedin G., Li G., Nord C.E. (2008). Genetic diversity of community-associated methicillin-resistant *Staphylococcus aureus* in southern Stockholm, 2000–2005. Clin. Microbiol. Infect..

[60] Petersson A.C., Olsson-Liljequist B., Miörner H., Haeggman S. (2010). Evaluating the usefulness of *spa* typing, in comparison with pulsed-field gel electrophoresis, for epidemiological typing of methicillin-resistant *Staphylococcus aureus* in a low-prevalence region in Sweden 2000-2004. Clin. Microbiol. Infect..

[61] Rossney A.S., Shore A.C., Morgan P.M., Fitzgibbon M.M., O’Connell B., Coleman D.C. (2007). The emergence and importation of diverse genotypes of methicillin-resistant *Staphylococcus aureus* (MRSA) harboring the Panton-Valentine leukocidin gene (*pvl*) reveal that *pvl* is a poor marker for community-acquired MRSA strains in Ireland. J. Clin. Microbiol..

[62] Shore A.C., Tecklenborg S.C., Brennan G.I., Ehricht R., Monecke S., Coleman D.C. (2014). Panton-Valentine leukocidin-positive *Staphylococcus aureus* in Ireland from 2002 to 2011: 21 clones, frequent importation of clones, temporal shifts of predominant methicillin-resistant *S. aureus* clones, and increasing multiresistance. J. Clin. Microbiol..

[63] Holmes A., Ganner M., McGuane S., Pitt T.L., Cookson B.D., Kearns A.M. (2005). *Staphylococcus aureus* isolates carrying Panton-Valentine leucocidin genes in England and Wales: Frequency, characterization, and association with clinical disease. J. Clin. Microbiol..

[64] Otter J.A., French G.L. (2008). The emergence of community-associated methicillin-resistant *Staphylococcus aureus* at a London teaching hospital, 2000–2006. Clin. Microbiol. Infect..

[65] Urth T., Juul G., Skov R., Schønheyder H.C. (2005). Spread of a methicillin-resistant *Staphylococcus aureus* ST80-IV clone in a Danish community. Infect. Control Hosp. Epidemiol..

[66] Bartels M.D., Boye K., Rhod Larsen A., Skov R., Westh H. (2007). Rapid increase of genetically diverse methicillin-resistant *Staphylococcus aureus*, Copenhagen, Denmark. Emerg. Infect. Dis..

[67] Bartels M.D., Larner-Svensson H., Meiniche H., Kristoffersen K., Schonning K., Nielsen J.B., Rohde S.M., Christensen L.B., Skibsted A.W., Jarlov J.O. (2015). Monitoring meticillin resistant *Staphylococcus aureus* and its spread in Copenhagen, Denmark, 2013, through routine whole genome sequencing. Euro Surveill..

[68] Larsen A.R., Stegger M., Böcher S., Sørum M., Monnet D.L., Skov R.L. (2009). Emergence and characterization of community-associated methicillin-resistant *Staphyloccocus aureus* infections in Denmark, 1999 to 2006. J. Clin. Microbiol..

[69] McLaws F.B., Larsen A.R., Skov R.L., Chopra I., O’Neill A.J. (2011). Distribution of fusidic acid resistance determinants in methicillin-resistant *Staphylococcus aureus*. Antimicrob. Agents Chemother..

[70] Vourli S., Perimeni D., Makri A., Polemis M., Voyiatzi A., Vatopoulos A. (2005). Community acquired MRSA infections in a paediatric population in Greece. Euro Surveill..

[71] Chini V., Petinaki E., Meugnier H., Foka A., Bes M., Etienne J., Dimitracopoulos G., Spiliopoulou I. (2008). Emergence of a new clone carrying Panton-Valentine leukocidin genes and staphylococcal cassette chromosome *mec* type V among methicillin-resistant *Staphylococcus aureus* in Greece. Scand. J. Infect. Dis..

[72] Vourli S., Vagiakou H., Ganteris G., Orfanidou M., Polemis M., Vatopoulos A., Malamou-Ladas H. (2009). High rates of community-acquired, Panton-Valentine leukocidin (PVL)-positive methicillin-resistant *S. aureus* (MRSA) infections in adult outpatients in Greece. Euro Surveill..

[73] Katopodis G.D., Grivea I.N., Tsantsaridou A.J., Pournaras S., Petinaki E., Syrogiannopoulos G.A. (2010). Fusidic acid and clindamycin resistance in community-associated, methicillin-resistant *Staphylococcus aureus* infections in children of Central Greece. BMC Infect. Dis..

[74] Hadjihannas L., Psichogiou M., Empel J., Kosmidis C., Goukos D., Bouzala J., Georgopoulos S., Malhotra-Kumar S., Harbarth S., Daikos G.L. (2012). Molecular characteristics of community-associated methicillin-resistant *Staphylococcus aureus* colonizing surgical patients in Greece. Diagn. Microbiol. Infect. Dis..

[75] Drougka E., Foka A., Liakopoulos A., Doudoulakakis A., Jelastopulu E., Chini V., Spiliopoulou A., Levidiotou S., Panagea T., Vogiatzi A. (2014). A 12-year survey of methicillin-resistant *Staphylococcus aureus* infections in Greece: ST80-IV epidemic?. Clin. Microbiol. Infect..

[76] Papadimitriou-Olivgeris M., Drougka E., Fligou F., Dodou V., Kolonitsiou F., Filos K.S., Anastassiou E.D., Petinaki E., Marangos M., Spiliopoulou I. (2017). Spread of *tst*–positive *Staphylococcus aureus* strains belonging to ST30 clone among patients and healthcare workers in two intensive care units. Toxins.

[77] Nikolaras G.P., Papaparaskevas J., Samarkos M., Tzouvelekis L.S., Psychogiou M., Pavlopoulou I., Goukos D., Polonyfi K., Pantazatou A., Deliolanis I. (2019). Changes in the Rates and Population Structure of MRSA from bloodstream infections. A Single Center Experience (2000–2015). J. Glob. Antimicrob. Resist..

[78] Aschbacher R., Pichon B., Spoladore G., Pagani E., Innocenti P., Moroder L., Ganner M., Hill R., Pike R., Ganthaler O. (2012). High clonal heterogeneity of Panton-Valentine leukocidin-positive meticillin-resistant *Staphylococcus aureus* strains from skin and soft-tissue infections in the Province of Bolzano, Northern Italy. Int. J. Antimicrob. Agents.

[79] Sanchini A., Campanile F., Monaco M., Cafiso V., Rasigade J.-P., Laurent F., Etienne J., Stefani S., Pantosti A. (2011). DNA microarray-based characterisation of Panton-Valentine leukocidin-positive community-acquired methicillin-resistant *Staphylococcus aureus* from Italy. Eur. J. Clin. Microbiol. Infect. Dis..

[80] Dermota U., Jurca T., Harlander T., Košir M., Zajc U., Golob M., Zdovc I., Košnik I.G. (2016). Infections caused by community-associated methicillin-resistant *Staphylococcus aureus* european clone (ST80) in Slovenia between 2006 and 2013. Zdr. Varst.

[81] Cercenado E., Cuevas O., Marín M., Bouza E., Trincado P., Boquete T., Padilla B., Vindel A. (2008). Community-acquired methicillin-resistant *Staphylococcus aureus* in Madrid, Spain: Transcontinental importation and polyclonal emergence of Panton-Valentine leukocidin-positive isolates. Diagn. Microbiol. Infect. Dis..

[82] Vindel A., Trincado P., Cuevas O., Ballesteros C., Bouza E., Cercenado E. (2014). Molecular epidemiology of community-associated methicillin-resistant *Staphylococcus aureus* in Spain: 2004–2012. J. Antimicrob. Chemother..

[83] Scicluna E.A., Shore A.C., Thürmer A., Ehricht R., Slickers P., Borg M.A., Coleman D.C., Monecke S. (2010). Characterisation of MRSA from Malta and the description of a Maltese epidemic MRSA strain. Eur. J. Clin. Microbiol. Infect. Dis..

[84] Conceição T., Aires-de-Sousa M., Pona N., Brito M.J., Barradas C., Coelho R., Sardinha T., Sancho L., de Sousa G., do Machado M.C. (2011). High prevalence of ST121 in community-associated methicillin-susceptible *Staphylococcus aureus* lineages responsible for skin and soft tissue infections in Portuguese children. Eur. J. Clin. Microbiol. Infect. Dis..

[85] Krziwanek K., Luger C., Sammer B., Stumvoll S., Stammler M., Metz-Gercek S., Mittermayer H. (2007). PVL-positive MRSA in Austria. Eur. J. Clin. Microbiol. Infect. Dis..

[86] Berktold M., Grif K., Mäser M., Witte W., Würzner R., Orth-Höller D. (2012). Genetic characterization of Panton-Valentine leukocidin-producing methicillin-resistant *Staphylococcus aureus* in Western Austria. Wien. Klin. Wochenschr..

[87] Denis O., Deplano A., De Beenhouwer H., Hallin M., Huysmans G., Garrino M.G., Glupczynski Y., Malaviolle X., Vergison A., Struelens M.J. (2005). Polyclonal emergence and importation of community-acquired methicillin-resistant *Staphylococcus aureus* strains harbouring Panton-Valentine leucocidin genes in Belgium. J. Antimicrob. Chemother..

[88] Brauner J., Hallin M., Deplano A., De Mendonça R., Nonhoff C., De Ryck R., Roisin S., Struelens M.J., Denis O. (2013). Community-acquired methicillin-resistant *Staphylococcus aureus* clones circulating in Belgium from 2005 to 2009: Changing epidemiology. Eur. J. Clin. Microbiol. Infect. Dis..

[89] Fenner L., Widmer A.F., Dangel M., Frei R. (2008). Distribution of *spa* types among meticillin-resistant *Staphylococcus aureus* isolates during a 6 year period at a low-prevalence University Hospital. J. Med. Microbiol..

[90] Wannet W.J.B., Spalburg E., Heck M.E.O.C., Pluister G.N., Tiemersma E., Willems R.J.L., Huijsdens X.W., de Neeling A.J., Etienne J. (2005). Emergence of virulent methicillin-resistant *Staphylococcus aureus* Strains carrying Panton-Valentine leucocidin genes in The Netherlands. J. Clin. Microbiol..

[91] Stam-Bolink E.M., Mithoe D., Baas W.H., Arends J.P., Möller A.V.M. (2007). Spread of a methicillin-resistant *Staphylococcus aureus* ST80 strain in the community of the northern Netherlands. Eur. J. Clin. Microbiol. Infect. Dis..

[92] Nulens E., Stobberingh E.E., Smeets E., van Dessel H., Welling M.A., Sebastian S., van Tiel F.H., Beisser P.S., Deurenberg R.H. (2009). Genetic diversity of methicillin-resistant *Staphylococcus aureus* in a tertiary hospital in The Netherlands between 2002 and 2006. Eur. J. Clin. Microbiol. Infect. Dis..

[93] Hetem D.J., Westh H., Boye K., Jarløv J.O., Bonten M.J.M., Bootsma M.C.J. (2012). Nosocomial transmission of community-associated methicillin-resistant *Staphylococcus aureus* in Danish Hospitals. J. Antimicrob. Chemother..

[94] Witte W., Pasemann B., Cuny C. (2007). Detection of low-level oxacillin resistance in *mecA*-positive *Staphylococcus aureus*. Clin. Microbiol. Infect..

[95] Witte W., Strommenger B., Cuny C., Heuck D., Nuebel U. (2007). Methicillin-resistant *Staphylococcus aureus* containing the Panton-Valentine leucocidin gene in Germany in 2005 and 2006. J. Antimicrob. Chemother..

[96] Monecke S., Jatzwauk L., Weber S., Slickers P., Ehricht R. (2008). DNA microarray-based genotyping of methicillin-resistant *Staphylococcus aureus* strains from Eastern Saxony. Clin. Microbiol. Infect..

[97] Schaumburg F., Köck R., Mellmann A., Richter L., Hasenberg F., Kriegeskorte A., Friedrich A.W., Gatermann S., Peters G., von Eiff C. (2012). Population Dynamics among Methicillin-Resistant *Staphylococcus aureus* Isolates in Germany during a 6-Year Period. J. Clin. Microbiol..

[98] Klein S., Menz M.-D., Zanger P., Heeg K., Nurjadi D. (2019). Increase in the prevalence of Panton-Valentine leukocidin and clonal shift in community-onset methicillin-resistant *Staphylococcus aureus* causing skin and soft-tissue infections in the Rhine-Neckar Region, Germany, 2012-2016. Int. J. Antimicrob. Agents.

[99] Dauwalder O., Lina G., Durand G., Bes M., Meugnier H., Jarlier V., Coignard B., Vandenesch F., Etienne J., Laurent F. (2008). Epidemiology of invasive methicillin-resistant *Staphylococcus aureus* clones collected in France in 2006 and 2007. J. Clin. Microbiol..

[100] Robert J., Tristan A., Cavalié L., Decousser J.-W., Bes M., Etienne J., Laurent F., ONERBA (Observatoire National de l’Epidémiologie de Résistance Bactérienne aux Antibiotiques) (2011). Panton-valentine leukocidin-positive and toxic shock syndrome toxin 1-positive methicillin-resistant *Staphylococcus aureus*: A French multicenter prospective study in 2008. Antimicrob. Agents Chemother..

[101] Maugat S., de Rougemont A., Aubry-Damon H., Reverdy M.-E., Georges S., Vandenesch F., Etienne J., Coignard B. (2009). Methicillin-resistant *Staphylococcus aureus* among a network of French private-sector community-based-medical laboratories. Med. Mal. Infect..

[102] Lamy B., Laurent F., Gallon O., Doucet-Populaire F., Etienne J., Decousser J.-W., Collège de Bactériologie Virologie Hygiène (ColBVH) Study Group (2012). Antibacterial resistance, genes encoding toxins and genetic background among *Staphylococcus aureus* isolated from community-acquired skin and soft tissue infections in France: A national prospective survey. Eur. J. Clin. Microbiol. Infect. Dis..

[103] Fluit A.C., Carpaij N., Majoor E.A.M., Weinstein R.A., Aroutcheva A., Rice T.W., Bonten M.J.M., Willems R.J.L. (2015). Comparison of an ST80 MRSA strain from the USA with European ST80 strains. J. Antimicrob. Chemother..

[104] Aung K.T., Hsu L.Y., Koh T.H., Hapuarachchi H.C., Chau M.L., Gutiérrez R.A., Ng L.C. (2017). Prevalence of methicillin-resistant *Staphylococcus aureus* (MRSA) in retail food in Singapore. Antimicrob. Resist. Infect. Control.

[105] Islam M.A., Parveen S., Rahman M., Huq M., Nabi A., Khan Z.U.M., Ahmed N., Wagenaar J.A. (2019). Occurrence and characterization of methicillin resistant *Staphylococcus aureus* in processed raw foods and ready-to-eat foods in an urban setting of a developing country. Front. Microbiol..

[106] Peeters L.E.J., Argudín M.A., Azadikhah S., Butaye P. (2015). Antimicrobial resistance and population structure of *Staphylococcus aureus* recovered from pigs farms. Vet. Microbiol..

[107] Agabou A., Ouchenane Z., Ngba Essebe C., Khemissi S., Chehboub M.T.E., Chehboub I.B., Sotto A., Dunyach-Remy C., Lavigne J.-P. (2017). Emergence of nasal carriage of ST80 and ST152 PVL+ *Staphylococcus aureus* isolates from livestock in Algeria. Toxins.

[108] Mairi A., Touati A., Pantel A., Zenati K., Martinez A.Y., Dunyach-Remy C., Sotto A., Lavigne J.-P. (2019). Distribution of toxinogenic methicillin-resistant and methicillin-susceptible *Staphylococcus aureus* from different ecological niches in Algeria. Toxins.

[109] Witte W. (2009). Community-acquired methicillin-resistant *Staphylococcus aureus*: What do we need to know?. Clin. Microbiol. Infect..

[110] Huijsdens X.W., van Lier A.M.C., van Kregten E., Verhoef L., van Santen-Verheuvel M.G., Spalburg E., Wannet W.J.B. (2006). Methicillin-resistant *Staphylococcus aureus* in Dutch soccer team. Emerg. Infect. Dis..

[111] Drougka E., Foka A., Koutinas C.K., Jelastopulu E., Giormezis N., Farmaki O., Sarrou S., Anastassiou E.D., Petinaki E., Spiliopoulou I. (2016). Interspecies spread of *Staphylococcus aureus* clones among companion animals and human close contacts in a veterinary teaching hospital. A cross-sectional study in Greece. Prev. Vet. Med..

[112] Kolonitsiou F., Papadimitriou-Olivgeris M., Spiliopoulou A., Drougka E., Jelastopulu E., Anastassiou E.D., Spiliopoulou I. (2018). Methicillin-resistant *Staphylococcus aureus* ST80 induce lower cytokine production by monocytes as compared to other Sequence Types. Front. Microbiol..

[113] Larsen A.R., Böcher S., Stegger M., Goering R., Pallesen L.V., Skov R. (2008). Epidemiology of european community-associated methicillin-resistant *Staphylococcus aureus* clonal complex 80 type IV strains isolated in Denmark from 1993 to 2004. J. Clin. Microbiol..

[114] Del Giudice P., Bes M., Hubiche T., Blanc V., Roudière L., Lina G., Vandenesch F., Etienne J. (2011). Panton-Valentine leukocidin-positive *Staphylococcus aureus* strains are associated with follicular skin infections. Dermatology.

[115] Harastani H.H., Tokajian S.T. (2014). Community-associated methicillin-resistant *Staphylococcus aureus* clonal complex 80 type IV (CC80-MRSA-IV) isolated from the Middle East: A heterogeneous expanding clonal lineage. PLoS ONE.

[116] Dunyach-Remy C., Ngba Essebe C., Sotto A., Lavigne J.-P. (2016). *Staphylococcus aureus* toxins and diabetic foot ulcers: Role in pathogenesis and interest in diagnosis. Toxins.

[117] Courjon J., Munro P., Benito Y., Visvikis O., Bouchiat C., Boyer L., Doye A., Lepidi H., Ghigo E., Lavigne J.-P. (2015). EDIN-B promotes the translocation of *Staphylococcus aureus* to the bloodstream in the course of pneumonia. Toxins.

[118] Gillet Y., Dumitrescu O., Tristan A., Dauwalder O., Jahouvey E., Floret D., Vandenesch F., Etienne J., Lina G. (2011). Pragmatic management of Panton-Valentine leukocidin-associated staphylococcal diseases. Int. J. Antimicrob. Agents.

[119] Zingg W., Holmes A., Dettenkofer M., Goetting T., Secci F., Clack L., Allegranzi B., Magiorakos A.P., Pittet D. (2015). Hospital organisation, management, and structure for prevention of health-care-associated infection: A systematic review and expert consensus. Lancet Infect. Dis..

[120] Al-Obaidi M.M.J., Suhaili Z., MohdDesa M.N. (2018). Genotyping Approaches for Identification and Characterization of Staphylococcus aureus.

[121] Boye K., Bartels M.D., Andersen I.S., Møller J.A., Westh H. (2007). A new multiplex PCR for easy screening of methicillin-resistant *Staphylococcus aureus* SCC*mec* types I-V. Clin. Microbiol. Infect..

[122] Liu J., Chen D., Peters B.M., Li L., Li B., Xu Z., Shirliff M.E. (2016). Staphylococcal chromosomal cassettes *mec* (SCC*mec*): A mobile genetic element in methicillin-resistant *Staphylococcus aureus*. Microb. Pathog..

[123] Rachman A.R.A., Suhaili Z., MohdDesa M.N. (2017). The evolution and dissemination of methicillin resistance determinant in *Staphylococcus aureus*. The Rise of Virulence and Antibiotic Resistance in Staphylococcus aureus.

[124] Rodriguez M., Hogan P.G., Satola S.W., Crispell E., Wylie T., Gao H., Sodergren E., Weinstock G.M., Burnham C.-A.D., Fritz S.A. (2015). Discriminatory indices of typing methods for epidemiologic analysis of contemporary *Staphylococcus aureus* strains. Medicine.

[125] Malachowa N., Sabat A., Gniadkowski M., Krzyszton-Russjan J., Empel J., Miedzobrodzki J., Kosowska-Shick K., Appelbaum P.C., Hryniewicz W. (2005). Comparison of multiple-locus variable-number tandem-repeat analysis with pulsed-field gel electrophoresis, spa typing, and multilocus sequence typing for clonal characterization of *Staphylococcus aureus* isolates. J. Clin. Microbiol..

[126] Alkharsah K.R., Rehman S., Alnimr A., Diab A., Hawwari A., Tokajian S. (2019). Molecular typing of MRSA isolates by *spa* and PFGE. J. King Saud Univ. Sci..

[127] Enright M.C., Day N.P.J., Davies C.E., Peacock S.J., Spratt B.G. (2000). Multilocus sequence typing for characterization of methicillin-resistant and methicillin-susceptible clones of *Staphylococcus aureus*. J. Clin. Microbiol..

[128] Urwin R., Maiden M.C.J. (2003). Multi-locus sequence typing: A tool for global epidemiology. Trends Microbiol..

[129] Boers S.A., van der Reijden W.A., Jansen R. (2012). High-throughput multilocus sequence typing: Bringing molecular typing to the next level. PLoS ONE.

[130] Hallin M., Deplano A., Denis O., De Mendonça R., De Ryck R., Struelens M.J. (2007). Validation of pulsed-field gel electrophoresis and *spa* typing for long-term, nationwide epidemiological surveillance studies of *Staphylococcus aureus* infections. J. Clin. Microbiol..

[131] Bumgarner R. (2013). DNA microarrays: Types, applications and their future. Curr. Protoc. Mol. Biol..

[132] Miao J., Chen L., Wang J., Wang W., Chen D., Li L., Li B., Deng Y., Xu Z. (2017). Current methodologies on genotyping for nosocomial pathogen methicillin-resistant *Staphylococcus aureus* (MRSA). Microb. Pathog..

[133] Kong Z., Zhao P., Liu H., Yu X., Qin Y., Su Z., Wang S., Xu H., Chen J. (2016). Whole-genome sequencing for the investigation of a hospital outbreak of MRSA in China. PLoS ONE.

[134] Quainoo S., Coolen J.P.M., van Hijum S.A.F.T., Huynen M.A., Melchers W.J.G., van Schaik W., Wertheim H.F.L. (2017). Whole-genome sequencing of bacterial pathogens: The future of nosocomial outbreak analysis. Clin. Microbiol. Rev..

[135] Schwarze K., Buchanan J., Taylor J.C., Wordsworth S. (2018). Are whole-exome and whole-genome sequencing approaches cost-effective? A systematic review of the literature. Genet. Med..

[136] Rolain J.-M., Abat C., Brouqui P., Raoult D. (2015). Worldwide decrease in methicillin-resistant *Staphylococcus aureus*: Do we understand something?. Clin. Microbiol. Infect..

[137] Walter J., Noll I., Feig M., Weiss B., Claus H., Werner G., Eckmanns T., Hermes J., Abu Sin M. (2017). Decline in the proportion of methicillin resistance among *Staphylococcus aureus* isolates from non-invasive samples and in outpatient settings, and changes in the co-resistance profiles: An analysis of data collected within the Antimicrobial Resistance Surveillance Network, Germany 2010 to 2015. BMC Infect. Dis..

[138] Lee A.S., de Lencastre H., Garau J., Kluytmans J., Malhotra-Kumar S., Peschel A., Harbarth S. (2018). Methicillin-resistant *Staphylococcus aureus*. Nat. Rev. Dis. Primers.

[139] Köck R., Becker K., Cookson B., van Gemert-Pijnen J.E., Harbarth S., Kluytmans J., Mielke M., Peters G., Skov R.L., Struelens M.J. (2010). Methicillin-resistant *Staphylococcus aureus* (MRSA): Burden of disease and control challenges in Europe. Euro Surveill..

[140] de Lencastre H., Severina E.P., Milch H., Thege M.K., Tomasz A. (1997). Wide geographic distribution of a unique methicillin-resistant *Staphylococcus aureus* clone in Hungarian hospitals. Clin. Microbiol. Infect..

[141] Oliveira D.C., Crisóstomo I., Santos-Sanches I., Major P., Alves C.R., Aires-de-Sousa M., Thege M.K., de Lencastre H. (2001). Comparison of DNA sequencing of the protein A gene polymorphic region with other molecular typing techniques for typing two epidemiologically diverse collections of methicillin-resistant *Staphylococcus aureus*. J. Clin. Microbiol..

[142] Conceição T., Aires-de-Sousa M., Füzi M., Tóth A., Pászti J., Ungvári E., van Leeuwen W.B., van Belkum A., Grundmann H., de Lencastre H. (2007). Replacement of methicillin-resistant *Staphylococcus aureus* clones in Hungary over time: A 10-year surveillance study. Clin. Microbiol. Infect..

[143] Asanin J., Misic D., Aksentijevic K., Tambur Z., Rakonjac B., Kovacevic I., Spergser J., Loncaric I. (2019). Genetic profiling and comparison of human and animal methicillin-resistant *Staphylococcus aureus* (MRSA) isolates from Serbia. Antibiotics.

[144] Monecke S., Gavier-Widén D., Hotzel H., Peters M., Guenther S., Lazaris A., Loncaric I., Müller E., Reissig A., Ruppelt-Lorz A. (2016). Diversity of *Staphylococcus aureus* isolates in european wildlife. PLoS ONE.

[145] Botelho A.M.N., Cerqueira E., Costa M.O., Moustafa A.M., Beltrame C.O., Ferreira F.A., Côrtes M.F., Costa B.S.S., Silva D.N.S., Bandeira P.T. (2019). Local diversification of methicillin- resistant *Staphylococcus aureus* ST239 in South America after its rapid worldwide dissemination. Front. Microbiol..

[146] Goudarzi M., Goudarzi H., Sá Figueiredo A.M., Udo E.E., Fazeli M., Asadzadeh M., Seyedjavadi S.S. (2016). Molecular characterization of methicillin resistant *Staphylococcus aureus* strains isolated from intensive care units in Iran: ST22-SCC*mec* IV/t790 emerges as the major clone. PLoS ONE.

[147] Goudarzi M., Eslami G., Rezaee R., Heidary M., Khoshnood S., Sajadi Nia R. (2019). Clonal dissemination of *Staphylococcus aureus* isolates causing nosocomial infections, Tehran, Iran. Iran. J. Basic Med. Sci..

[148] Goudarzi M., Kobayashi N., Hashemi A., Fazeli M., Navidinia M. (2019). Genetic variability of methicillin resistant *Staphylococcus aureus* strains isolated from burns patients. Osong Public Health Res. Perspect..

[149] Çakıcı N., Akçalı A., Demirel Zorba N.N. (2019). Antibiotic resistance pattern and *spa* types of *Staphylococcus aureus* strains isolated from food business and hospital kitchen employees in Çanakkale, Turkey. Turk. J. Med. Sci..

[150] Chang Q., Abuelaish I., Biber A., Jaber H., Callendrello A., Andam C.P., Regev-Yochay G., Hanage W.P., On Behalf of the PICR Study Group (2018). Genomic epidemiology of meticillin-resistant *Staphylococcus aureus* ST22 widespread in communities of the Gaza Strip, 2009. Euro Surveill..

[151] Mobasherizadeh S., Shojaei H., Azadi D., Havaei S.A., Rostami S. (2019). Molecular characterization and genotyping of methicillin-resistant *Staphylococcus aureus* in nasal carriage of healthy Iranian children. J. Med. Microbiol..

[152] Sobhanipoor M.H., Ahmadrajabi R., Karmostaji A., Saffari F. (2017). Molecular characterization of nasal methicillin resistant *Staphylococcus aureus* isolates from workers of an automaker company in southeast Iran. APMIS.

[153] Udo E.E., Boswihi S.S., Al-Sweih N. (2016). High prevalence of toxic shock syndrome toxin-producing epidemic methicillin-resistant *Staphylococcus aureus* 15 (EMRSA-15) strains in Kuwait hospitals. New Microbes New Infect..

[154] Goudarzi M., Fazeli M., Pouriran R., Eslami G. (2019). Genotype distribution of Panton-Valentine leukocidin (PVL)-positive *Staphylococcus aureus* strains isolated from wound-related infections: A three-year multi-center study in Tehran, Iran. Jpn. J. Infect. Dis..

[155] Conceição T., de Lencastre H., Aires-de-Sousa M. (2017). Carriage of *Staphylococcus aureus* among Portuguese nursing students: A longitudinal cohort study over four years of education. PLoS ONE.

[156] Groves M.D., Crouch B., Coombs G.W., Jordan D., Pang S., Barton M.D., Giffard P., Abraham S., Trott D.J. (2016). Molecular epidemiology of methicillin-resistant *Staphylococcus aureus* isolated from Australian veterinarians. PLoS ONE.

[157] Rodrigues A.C., Belas A., Marques C., Cruz L., Gama L.T., Pomba C. (2018). Risk Factors for nasal colonization by methicillin-resistant staphylococci in healthy humans in professional daily contact with companion animals in Portugal. Microb. Drug Resist..

[158] Rodríguez-Lázaro D., Oniciuc E.-A., García P.G., Gallego D., Fernández-Natal I., Dominguez-Gil M., Eiros-Bouza J.M., Wagner M., Nicolau A.I., Hernández M. (2017). Detection and characterization of *Staphylococcus aureus* and methicillin-resistant *S. aureus* in foods confiscated in EU borders. Front. Microbiol..

[159] Tosas Auguet O., Stabler R.A., Betley J., Preston M.D., Dhaliwal M., Gaunt M., Ioannou A., Desai N., Karadag T., Batra R. (2018). Frequent undetected ward-based methicillin-resistant *Staphylococcus aureus* transmission linked to patient sharing between hospitals. Clin. Infect. Dis..

[160] Worthing K.A., Abraham S., Pang S., Coombs G.W., Saputra S., Jordan D., Wong H.S., Abraham R.J., Trott D.J., Norris J.M. (2018). Molecular characterization of methicillin-resistant *Staphylococcus aureus* isolated from Australian animals and veterinarians. Microb. Drug Resist..

[161] Gostev V., Kruglov A., Kalinogorskaya O., Dmitrenko O., Khokhlova O., Yamamoto T., Lobzin Y., Ryabchenko I., Sidorenko S. (2017). Molecular epidemiology and antibiotic resistance of methicillin-resistant *Staphylococcus aureus* circulating in the Russian Federation. Infect. Genet. Evol..

[162] Htun H.L., Kyaw W.M., de Sessions P.F., Low L., Hibberd M.L., Chow A., Leo Y.S. (2018). Methicillin-resistant *Staphylococcus aureus* colonisation: Epidemiological and molecular characteristics in an acute-care tertiary hospital in Singapore. Epidemiol. Infect..

[163] Xiao N., Yang J., Duan N., Lu B., Wang L. (2019). Community-associated Staphylococcus aureus PVL+ ST22 predominates in skin and soft tissue infections in Beijing, China. Infect. Drug Resist..

