# Supplementary material for: Methicillin-Resistant Staphylococcus aureus ST80 Clone: A Systematic Review
Source: Toxins (Basel). 2020 Feb 14;12(2):119. doi: 10.3390/toxins12020119 (PMC7076798; doi:10.3390/toxins12020119)
Supplement: Supplementary file 1 [file toxins-12-00119-s001.pdf]

Supplementary Materials: Methicillin-Resistant *Staphylococcus aureus* ST80 clone: a systematic review

Assia Mairi, Abdelaziz Touati and Jean-Philippe Lavigne *

Table S1. Characteristics of MRSA-ST80 isolates published and studied in this review.

Country	Period of sampling	Number of *S. aureus* isolates	Number of MRSA isolates	Number of MRSA-ST80 isolates	Number of MRSA-ST80 PVL+ isolates	Type of samples	Clinical details	Other toxins associated with MRSA-ST80	*spa*-typing	Antimicrobial resistance	Comments	Reference
**Multicenter studies**												
17 countries in the Americas, Australia-Asia and Europe	2009–2010	ND	3236 PVL-positive MRSA	ND (MRSA-ST80 were reported in Europe and were not reported from other regions)	ND	Patients	ND	ND	ND	-	Retrospective laboratory-based survey	[1]
16 European countries (Spain, France, Poland,	2000–2010	568 (67 HA-*S. aureus* and 145 CA-*S.*	127 (43 HA-MRSA and 84	51	48	Patients	Clinical samples	ND	t044, t067, t131, t376	-	Multicenter study (health-care centers and hospitals)	[2]

Bulgaria, Czech Republic, Netherlands, Greece, Denmark, Finland, Slovakia, Italy, Hungary, Sweden, United Kingdom, Romania, and Portugal)		*aureus* were analyzed)	CA-MRSA)									
						**North Africa**						
	2004–2007	843	23	15	15	Inpatient (10), outpatient (5)	Infections	ND	t044	ND	Hospital	[3]
Algeria	2006–2007	221	99 (34 CA-MRSA and 65 HA-MRSA)	35.7%	-	Patients	SSTIs	*etD*, *edin*	t044	ND	Hospital	[4]
	2010–2011	129	25 (15 CA-MRSA and 10	24	22	CA-MRSA (9), HA-MRSA (15)	Infections	ND	ND	TE (19), CIP (6), ERY (7), CHL (8), DA (3)	Neonates and children	[5]

		HA-MRSA)									
2011–2012	85	73	10	10	Patients	Diabetic foot infections	*etD, edinB*	ND	ND	Diabetic patients	[6]
2010	177	92	19	19	7 CA-MRSA and 12 HA-MRSA	SSTIs (7)	ND	t044	KAN (19), ERY (2), LIN (2), TE (2)	Hospital	[7]
2010–2012	159	9	4	3	Surgery (2) and nephrology (2)	Nasal carriage	ND	ND	TOB (2), GEN (2), TE (1), ERY (1)	General hospital and hemodialysis ward	[8]
2015–2016	118	6	6	6	Camels (2) and sheep (4)	Nasal carriage	*etD, edinB, hla, hld, hlgA, hlg, clfA, clfB, fnbA, fnbB, cap8, sak, scn*	ND	KAN [*aphA3*]	Livestock	[9]
2018	312	20	20	20	Different ecological niches	Nasal and fecal swabs	*lukD, lukE* (19), *lukX* (7),*lukY* (15), *etD, edinB, hla* (10), *hlb* (15), *hld, hlgA, hlg, ebpS, bbp,*	ND	FOS (1), KAN, RIF, ERY (3) [*blaZ* (2), *ermC* (3), *aphA3*, *sat*, *fos* (1)]	Humans (3), pets (2), livestock (8), wild animals (4), and food (3)	[10]

								fib, clfA, clfB, fnbA, fnbB, sak, scn (17), cap8				
Tunisia	2008–2009	55	1	1	1	Veterinarian	Nasal swabs	ND	t203	TE, SMN, KAN [*tetK, ant(6)-Ia, aph(3′)-IIIa*]	Humans in contact with animals/no contact with animals	[11]
	2003–2005	ND	64	64	64	Outpatients	Infections	*etD, edinB, hlb*	t044	AK (64), KAN (63), ERY (30), TE (3), FUS (2)	-	[12]
	2013	ND	8	8	8	Children	Infections	ND	ND	KAN (8), ERY (2)	Hospitalized children	[13]
Libya	2008–2014	ND	95	17	17	Patients	Clinical samples	ND	t044	ND	Admitted and hospitalized patients (Medical center)	[14]
Egypt	2007	ND	21	2	2	Patients	Pathological samples	*hla, hld, hlg, hlgv, set, lukE, lukD, etD, eno, fnbA, clfA, clfB, fib, ebpS, icaA, icaD*	t044, t042	KAN (2), STM (2), DA (1)	Private clinic	[15]

**Middle East**												
Jordan	2008	103	41	4	4	Patients	Nose and stools	ND	t044	ND	Hospital	[16]
	2009	ND	37	2	2	Volunteer	Nasal and skin swabs	*hla*, *tst-1*, *seb*	t044	ND	Students and employees	[17]
	2009–2010	60	41	14	14	Patients	Infections	ND	t044	ND	Hospital	[18]
Saudi Arabia	2010–2011	ND	106	37	33	Patients	Carrier (19) and infections (18)	*tst-1* (2)	ND	ND	Hospital	[19]
	2010–2011	ND	58	21	19	Patients	Nasal swabs (2) and infections (19)	*lukD/E* (21), *seb/sek/seq*, (1), *edinB* (21), *etD* (21), *sak/scn/chp* (20), *cap8* (21)	ND	ERY (4), DA (4) [*ermC* (6/21), *aphA3/sat* (17/21), *far1* (18/21), *tetK* (2/21)]	Hospital	[20]
Saudi Arabia and Egypt	2011	104	59	3	3	Outpatients	Nasal swabs	ND	t044	ND	Community	[21]
Qatar	2009–2010	ND	61	8	8	Patients	Nasal swab (1) and infections (7)	ND	t044	ND	Hospital	[22]
Kuwait	2001–2003	ND	26 (7 outpatients and 19	10	10	Patients	Infections	*cap*8	ND	KAN (7), TE (8), FUS (10), DA (9)	Hospital	[23]

			inpatients)								
	2005–2006	ND	889 (135 CA-MRSA)	69	58	Patients	Infections and colonization	*sei* (54), *seg* (25), *sed* (19), *tst-1* (1), *cap*8 (69)	ND	KAN (23), TE (23), FUS (23), CIP (18), GEN (5), ERY (11), CHL (2), TMP (3), MUP (1)	Hospital	[24]
	2007	ND	4	4	4	Diabetic patient	Clinical samples	*cap8*	ND	KAN, TE, FUS, MUP, SMN [*tetK*, *far1*, *mupA*]	Hospital (4 MRSA in the same patient)	[25]
	1992–2010	ND	400	30	28	Patients	Clinicals amples	*etD* (30), *seb* (1), *sek* (1), *seq* (1)	t044 (24), t376 (1), t8154 (1), t1154 (1), tt018 (1), t042 (1), t852 (1)	KAN (30), TE (30), FUS (30), ERY (2), DA (2/30) [*aphA3* (30), *far1* (30), *sat* (30), *ermC*(2), *tetK* (2)]	13 public hospitals	[26]
	2005–2007	ND	25 (acquired in the ICU)	3	3	Patients	Infections and nasal sample	ND	t044 (2), t5393 (1)	KAN (3), FUS (2), SMN (2)	Hospital	[27]
	2006–2011	ND	103 (72 MRSA in neonates, 18 MRSA in adult	8	7	3 MRSA in neonates, 3 MRSA in adult	Infections	ND	t044 (7), t376 (1)	KAN (4), SMN (6), TE (4), FUS (4), TMP (3),	Maternity Hospital	[28]

			patients and 13 MRSA in healthcare workers)			patients and 2 MRSA in healthcare workers				ERY (2), DA (2)		
Lebanon	2006–2007	130	93	41	40	Patients	Skin sources	ND	t044 (40), t131 (1)	ND	Hospital	[29]
	2011	132	39	10	9	Patients		ND	t044 (5), t021 (1), t131 (3), t6476 (1)	ND	Hospital	[30]
Lebanon and Jordan	2000–2011	478	94	23	17	Patients	Clinical samples	*etA* (1), *etD* (21), *tst-1* (6)	t044 (13), t131 (3), t4222 (1), t5849 (2), t5802 (1), t6438 (1), t021 (1), t9135 (1)	KAN (21), TE (6), FUS (21), ERY (8), SMN (22), DA (2)	Hospital	[31]
Iran	-	38	17	1	1	Healthcare workers	Nasal swab	ND	t416	TE, GEN, SXT, CIP, RIF	Hospital	[32]
	2015–2017	240	70 (13 CA-MRSA and 57 HA-MRSA)	5 (1 CA-MRSA and 4 HA-MRSA)	5	Patients	Clinical samples	*clfB* (5), *clfA* (1), *fnbB* (3), *fnbA* (4), *ebp* (1), *bbp* (3), *cna* (2)	t044	AK (4), TMN (3), GEN (1), TE (2), CIP (4), ERY (5), RIF (4), DA (1) [*ermC* (2), *tetM* (2), *ant(4')-Ia* (2),	Four Hospitals	[33]

										aac(6′)-Ie/aph(2″) (1), *aph(3′)-IIIa* (2)]		
United Arab Emirates	2003 and 2008	ND	52	10	10	Patients	Clinical samples	*seb* (1)	t044	KAN (5), FUS (7), TE (4), ERY (1), DA (3), SMN (9), CIP (1)	Hospital	[34]
Turkey	2007–2012	ND	102	3	3	Patients	Infections	ND	ND	KAN, FUS	Hospital	[35]
Oman	2011	ND	79	3	3	Patients	Infections	ND	t044	KAN (3), GEN (1), SMN (3), TE (3), FUS (3), DA (1)	Hospital	[36]
Palestine	2008–2012	215	121	37	2	Patients	Infections	*tst-1* (1)	t044 (36), t458 (1)	ND	Hospital	[37]
						**Asia**						
Singapore	2011–2013	227	5	3	0	Retail foods	Samples (onion, fritters and egg)	*etD, seb, sek*	t1198	-	-	[38]
Bangladesh	2015–2016	430	132	5	0	Patients	Skin and soft tissue infections	*lukDE* (5), *tst -1* (1), *etD* (5), *edinB* (5), *fnbB* (5), *sea* (4), *sed* (1), *sec* (1), *she* (5), *sei* (1), *sej* (1), *sek*	t044 (1), t8154 (1), t8731 (1), t1193 (2)	ERY	Hospital	[39]

								(5), *sel* (1), *sen* (1), *seo* (1), *seq* (5), *selx* (1), *sely* (4)				
	2010–2013	35	9	3	2	Retail foods	Samples (meatball, burger and sweets)	*seb*	t8731 (1), t1198 (2)	ERY	-	[40]
Malaysia	2003–2008	ND	154	1	ND	Dialysis patient	Nasal swab	ND	t044	ND	Hospital	[41]
**Europe**												
Greece	1993 and 1998–2000	ND	118	11	11	Inpatients	Infections	ND	ND	ERY (1), FUS (10)	Hospital	[42]
	2004	ND	20 (CA-MRSA)	18	18	Patients	SSTI	ND	ND	TE, KAN, FUS	Pediatric hospital	[43]
	2004–2005	770	694 (382 CA-MRSA and 312 HA-MRSA)	428	428	Patients	Infections	ND	ND	FUS (418), KAN (428), ERY (6), DA (6)	Hospital	[44]
	2006–2007	88	27	27	27	Patients	Skin and soft tissue infections	ND	t044 (26), t131 (1)	TE, KAN, FUS	Hospital	[45]
	2003–2009	309	180 (CA-MRSA)	61	54/56 MRSA-ST80 tested	Children	Infections	ND	ND	FUS (58), TE (48), ERY (11), DA (16)	Outpatient clinics and hospital	[46]

2009	ND	51	7	7	Patients	Nasal swabs	ND	t044 (6), t131 (1)	CIP (1), ERY (2), DA (2), FUS (7), TE (7)	Hospital	[47]
2010	1	1	1	1	A 28-day-old boy	Orbital cellulitis (culture of pus)	ND	ND	TE, FUS	Hospital	[48]
2001–2012	ND	4614	2838 (2520 CA-MRSA and 318 HA-MRSA)	2825	Patients	SSTI and other infections	*egc* (222)	ND	-	Six hospitals	[49]
2007–2014	41	31 (20 MRSA were selected)	19	19	Children	Pneumonia	*fnbA*	ND	-	Hospital	[50]
2012–2013	60 (53 from pets and seven from veterinary personnel)	16	5	5	Pets and veterinary personnel	Nasal swabs	ND	ND	TE (4), FUS (4), KAN (4)	Companion animal clinics	[51]
2010–2011	143	57 (35 CA-MRSA and 22	45 (33 CA-MRSA and 12	45	Patients and healthcare workers	Infections and colonization	ND	ND	-	Hospital	[52]

			HA-MRSA)	HA-MRSA)								
	2000–2015	ND	398	19	19	Patients	Bloodstream infections	ND	t044	TE (11), FUS (11)	Hospital	[53]
Sweden	2000–2005	ND	367 (104 CA-MRSA)	35 (CA-MRSA)	35	Patients	Infections	ND	ND	ND	Hospital (4 MRSA-ST80 family-transmitted)	[54]
	2000–2004	ND	216	36	36	Patients	Clinical samples	ND	t042 (1), t044 (31), t131 (4)	ND	Hospital	[55]
Denmark	1997–2003	ND	118	118	118	Patients	Clinical samples	ND	ND	TE, KAN, FUS	Hospitals	[56]
	2001	ND	81 (36 HA-MRSA and 45 CA-MRSA)	38 (33 CA-MRSA and 5 HA-MRSA)	38	Patients	Infections	ND	ND	KMN	National Reference Centre for *Staphylococci*	[57]
	2003–2004	ND	143 (42 HA-MRSA and 101 CA-MRSA)	19 (18 CA-MRSA and 1 HA-MRSA)	19	Patients	Colonization	ND	t044 (16), t376 (2), t455 (1)	-	Five hospitals	[58]
	1999–2006	ND	2692 (studied only 526 CA-MRSA	206	65/65 (tested only 65 MRSA-ST80)	Patients	Infections	ND	t042, t044, t131, t376, t455, t1109	TE (175), FUS (191), RIF (1), STM (175), KAN (187),	14 regional laboratories processing all microbiologi	[59]

			isolated from infections)							ERY (11), DA (9)	cal specimens from hospitals, outpatient clinics, and general practitioners	
	2003–2005	ND	1639 (291 MRSA FUS-resistant were studied)	178 (only 6 isolates were studied)	ND	Patients	ND	ND	ND	[*fusB* (5)]	Hospital	[60]
	2010–2013	ND	341	13	13	Patients	Infections and colonization	ND	ND	t044 (7), t131 (1), t376 (3), t1028 (2)	Hospitals	[61]
	1993–2015	217 CC80 *S. aureus* (13 MSSA and 204 MRSA)	204	201	185	Patients	SSTI, other infections and carriage	ND	t044 (138), t131 (8), t376 (6), t934 (4), t1200 (10), t1028 (19), t1198 (1), t1247 (4), t4152 (1), t5088 (2), t5627 (1), t5849 (1), t5941 (1),	[*fusB* (159), *tetK* (146), *aadK/aphA* (133)]	Hospitals	[62]

									t6467 (2), t8731 (3)			
Denmark, Egypt, Lebanon	1997–2003	ND	ND	6	6	Patients	Infections	ND	t044	KAN (4), TE (5), FUS (6), STM (3), DA (1)	Hospital	[63]
Norway	1995–2003	ND	143 (67 MRSA were studied)	13	13	Inpatients and outpatients	Nasal swab (2) and infections (11)	ND	ND	KAN, STM	11 hospitals	[64]
	1991–2003	ND	110	27	ND	Patients	Infections	ND	t044	ND	Hospitals	[65]
	2011	ND	179 PVL-positive MRSA	34	34	Patients	Infections	*etD, edinB*	t044	(*aphA3, sat, tetK, far1*)	Hospital	[66]
France	2006–2007	ND	105	4	4	Patients	Invasive sampling of blood	*etD, edin*	t044	KAN (4), FUS (4), ERY (1)	23 hospitals	[67]
	2003	ND	238 (166 HA-MRSA, 39 CA-MRSA, 14 Nursing-associated-MRSA), and 64	1 (HA-MRSA)	1	Outpatients	Skin and soft tissue	ND	ND	-	45 private-sector community-based-medical laboratories	[68]

			unknown)									
	1999–2009	ND	20	20	20	Patients	Skin infections	*etD*, *edin*	t044	TE, KAN, FUS	Hospital	[69]
	2008	34970	7253 (333 MRSA were analyzed)	91 (CA-MRSA)	89	Patients	Clinical samples	*edin*(89), *etD* (89)	t044	TE (18), ERY (64), RIF (91)	104 laboratories	[70]
	2006	235	34 (CA-MRSA)	1	1	Patients	SSTI	ND	ND	KAN, FUS	71 non-teaching hospitals	[71]
	2008–2013	1283	77	15	12	Pediatric inpatients	Infections	ND	ND	TE (6), KAN (12), ERY (1)	Three hospitals	[72]
Germany	2003–2004	ND	8	8	8	8 patients	Infections and nasal swabs	*lukF/S/D/E*, *hlgA*, *hla*, *hlb*, *hld*, *etD*, *edinB*	t044 (7), t131 (1)	TE (7), FUS (7), STM (8), NMN (8) [*aphA3* (8), *sat* (8), *far1* (7), *tetK* (7)]	Hospitals	[73]
	2004–2006	100 (30 *S. aureus*-PVL+)	3	2	2	Patients	SSTI	*etD*, *edinB*	ND	ND	Hospital	[74]
	2005	ND	2125 (74 low-level oxacillin-resistant isolates were of particula	33 (CA-MRSA)	ND	Patients	Nasal colonization and various types of infections	ND	ND	ND	National Reference Centre for *Staphylococci*	[75]

			r interest)									
	2005–2006	ND	4815 (117 MRSA PVL+ were analyzed)	80 (CA-MRSA)	80	Patients	SSTI (73), pneumonia (1), septicemia (1), nasal colonization (5)	ND	t044	ND	German National Reference Laboratory for typing	[76]
	2000–2007	ND	80	8	8	Patients	Clinical samples	*etD, edinB, lukD/E/F/S, hl, hla, hlgA, hlb, hld, sak, scn, cap8, clfA, clfB, ebh, ebpS, eno, fib*	t044 or t131 (Microarray-based genotyping)	[*aphA3, sat, far1, tetK*]	Healthcare facilities, including a university hospital, a long-term rehabilitation facility and four hospitals.	[77]
	2004–2005 and 2010–2011	ND	3207	10	10	Patients	Infections	ND	t044	ND	36 microbiological laboratories throughout Germany	[78]
	2012–2016	ND	94	6	6	Patients	SSTI	ND	t044	ND	Hospital	[79]
United Kingdom	2002	515	15	12	12	Patients	Infections	*etD*	ND	TE (8), ERY (2), CHL (2), FUS (9), CIP (1)	Hospital	[80]

United Kingdom	2000–2006	ND	450 (CIP-S)	12 (3 HA-MRSA and 9 CA-MRSA)	12	Inpatient (4) and outpatient (8)	SSTI	ND	t044	ERY (6), FUS (6), TE (5)	Hospital	[81]
Belgium	2002–2004	ND	41 (16 MRSA PVL+ were analyzed)	9	9	Patients	Clinical samples	ND	t044 (8), t131 (1)	KAN (7), TE (6), FUS (8)	Reference laboratory for *Staphylococci*	[82]
	2005–2009	ND	410 (159 CA-MRSA PVL+ were studied)	76	76	Ambulatory patients	Clinical samples	*etD* (73), *seg* (1), *sen* (1)	t044	KAN (74), TE (69), ERY (15), FUS (65), CIP (1) [*aphA3* (74), *ant4* (1), *tetK* (69), *ermC* (15), *far1* (65)]	Reference laboratory for *Staphylococci* (74 Belgian microbiology laboratories)	[83]
	2013	ND	215	2	2	Pig farms	Nasal swabs	*fnbB, edinB, sasG, sak, scn, hla, hld, hIII, lukF/S/E/D, seg, sei, selm, selo, selu, etD, cap8*	t044	-	328 Farms	[84]
Spain	2004–2007	ND	53 (13 MRSA PVL+)	1	1	Patient	Infections	ND	ND	TE, FUS	Hospital	[85]

	2004–2012	ND	8326 (246 CA-MRSA were analyzed)	5	5	Patients	Infections	ND	t044 (4), t131(1)	-	Spain Reference laboratory for *Staphylococci*	[86]
Italy	2005–2009	ND	18 (CA-MRSA)	4	4	Patients	Necrotising pneumonia (2), SSTI (2)	*lukS/F/E/D/X/Y, hlgA, hla, hld, hlb, sak, scn, etD, edinB, bbp, clfA, clfB, ebh, ebpS, eno, fib, fnbA, fnbB, map, cap8*	t044 (3), t2453 (1)	[*ermC* (1), *aphA3, sat, far1, tetK*]	Hospitals	[87]
	2010–2011	ND	10 MRSA PVL+	1	1	Outpatient	Furunculosis	*etD, seh*	t044	CIP	Hospital	[88]
Ireland	2003	ND	1389 (25 MRSA PVL+)	2 (1 CA-MRSA and 1 HA-MRSA)	2	Patients	Colonization (1) and blood stream infection (1)	*etD*	t044	FUS (2), KAN (2), NMN (2), STM (2), TE (2), CHL (1)	23 Irish hospitals	[89]
	2002–2011	7103 (229 *S. aureus* PVL+ were analyzed)	190	27	27	Patients	Infections	*etD, edin*	t044 (21), t376 (5), t131 (1)	KAN (21), NEO (21), TE (21), FUS (20), CIP (1), ERY (11), DA (23), CHL (1), TMP (1)	Irish National MRSA Reference Laboratory	[90]

										[*tetK* (19), *aphA3* (29), *sat* (29), *fusB* (29), *ermC* (11)]		
Netherlands	1987–1995 and 2000–2002	ND	413 MRSA (20 MRSA PVL+ were studied)	12	12	Patients	SSTI	*etD*, *edin*	ND	ND	Hospital	[91]
	2005–2006	ND	12 (CA-MRSA)	12	12	Dutch soccer team	SSTI	ND	t044	TE, FUS, KAN	-	[92]
	1998–2005	ND	54 MRSA PVL+ were studied	43	43	Community (32), hospitals (4) and nursing homes (7)	SSTI (28), colonization (9), others (6)	ND	ND	ND	-	[93]
	2002–2006	ND	175	2	2	Patients	Clinical samples	ND	t044, t131	ND	Hospital	[94]
	2003–2010	ND	117	3 (CA-MRSA)	3	Patients	Infections	ND	t044	ND	Four hospitals	[95]
Austria	2001–2006	ND	1150 (94 MRSA PVL+ were analyzed)	14	14	Patients	Infections	ND	ND	ND	50 medical facilities (hospitals, medical doctors, private laboratories)	[96]

	2005–2010	ND	650 (31 MRSA PVL+ were studied)	3	3	Patients	SSTI	ND	t044, t455, t1201	FUS (2), ERY (1)	Hospitals	[97]
Switzerland	2000–2005	ND	200	7	3	Patients	Clinical samples	*etD* (3/7)	t044	ND	Hospital	[98]
Croatia	2004	1815	248	8	3	Patients	Clinical samples	ND	t044, t1605, t2608	ND	24 hospitals	[99]
Malta	2008–2009	ND	45	1	1	Hospital employee	Wound swab	*lukF/S/D/E, etD, hlgA, hl, hla, hlb, hld, edinB, ebh, bbp, clfA, clfB, eno, fib, fnbA, fnbB*	ND	STM, NEO, FUS, TE, KAN [*aphA3, sat, far1, tetK*]	Hospital	[100]
Portugal	2005–2006	38	3	1	1	Children	SSTI	*lukDE, hlg, etD, sel*	t044	ND	Hospital	[101]
Slovenia	2006–2013	ND	385 (CA-MRSA)	2	2	Patients	SSTI (1), sepsis and meningitis (1)	*etD*	t044	TE, FUS, KAN,	Hospital	[102]
North America												
USA	2006	ND	ND	1	1	Patient	Urinary tract infection			GEN, TOB, DA, ERY, CIP, TE, FUS	Hospital	[103]

AK: amikacin, CHL: chloramphenicol, CIP: ciprofloxacin, DA: clindamycin, ERY: erythromycin, FUS: fusidic acid, GEN: gentamicin, KAN: kanamycin, MUP: mupirocin, NEO: neomycin, NMN: netilmicin, RIF: rifampicin, SSTI: skin and soft tissue infections, STM: streptomycin, TE: tetracycline, TOB: tobramycin, TMP: trimethoprim, ND:

not determined, MRSA: Methicillin resistant S. aureus, CA-MRSA: Community-acquired MRSA, HA-MRSA: Hospital-acquired MRSA, PVL: Panton Valentine Leukocidin, ICU, intensive care unit.

References

1. Macedo-Viñas, M.; Conly, J.; Francois, P.; Aschbacher, R.; Blanc, D.S.; Coombs, G.; Daikos, G.; Dhawan, B.; Empel, J.; Etienne, J.; et al. Antibiotic susceptibility and molecular epidemiology of Panton-Valentine leukocidin-positive meticillin-resistant *Staphylococcus aureus*: An international survey. *J. Glob. Antimicrob. Resist.* **2014**, *2*, 43–47.
2. Rolo, J.; Miragaia, M.; Turlej-Rogacka, A.; Empel, J.; Bouchami, O.; Faria, N.A.; Tavares, A.; Hryniewicz, W.; Fluit, A.C.; de Lencastre, H.; et al. High genetic diversity among community-associated *Staphylococcus aureus* in Europe: Results from a multicenter study. *PLoS ONE* **2012**, *7*, e34768.
3. Bekkhoucha, S.N.; Cady, A.; Gautier, P.; Itim, F.; Donnio, P.-Y. A portrait of *Staphylococcus aureus* from the other side of the Mediterranean Sea: Molecular characteristics of isolates from Western Algeria. *Eur. J. Clin. Microbiol. Infect. Dis.* **2009**, *28*, 553–555.
4. Antri, K.; Rouzic, N.; Dauwalder, O.; Boubekri, I.; Bes, M.; Lina, G.; Vandenesch, F.; Tazir, M.; Ramdani-Bouguessa, N.; Etienne, J. High prevalence of methicillin-resistant *Staphylococcus aureus* clone ST80-IV in hospital and community settings in Algiers. *Clin. Microbiol. Infect.* **2011**, *17*, 526–532.
5. Djoudi, F.; Bonura, C.; Benallaoua, S.; Touati, A.; Touati, D.; Aleo, A.; Cala, C.; Fasciana, T.; Mammina, C. Panton-Valentine leukocidin positive sequence type 80 methicillin-resistant *Staphylococcus aureus* carrying a *Staphylococcal* cassette chromosome *mec* type IVc is dominant in neonates and children in an Algiers hospital. *New Microbiol.* **2013**, *36*, 49–55.
6. Djahmi, N.; Messad, N.; Nedjai, S.; Moussaoui, A.; Mazouz, D.; Richard, J.-L.; Sotto, A.; Lavigne, J.-P. Molecular epidemiology of *Staphylococcus aureus* strains isolated from inpatients with infected diabetic foot ulcers in an Algerian University Hospital. *Clin. Microbiol. Infect.* **2013**, *19*, E398–E404.
7. Alioua, M.A.; Labid, A.; Amoura, K.; Bertine, M.; Gacemi-Kirane, D.; Dekhil, M. Emergence of the European ST80 clone of community-associated methicillin-resistant *Staphylococcus aureus* as a cause of healthcare-associated infections in Eastern Algeria. *Med. Mal. Infect.* **2014**, *44*, 180–183.
8. Djoudi, F.; Benallaoua, S.; Aleo, A.; Touati, A.; Challal, M.; Bonura, C.; Mammina, C. Descriptive epidemiology of nasal carriage of *Staphylococcus aureus* and methicillin-resistant *Staphylococcus aureus* among patients admitted to two healthcare facilities in Algeria. *Microb. Drug Resist.* **2015**, *21*, 218–223.
9. Agabou, A.; Ouchenane, Z.; Ngba Essebe, C.; Khemissi, S.; Chehboub, M.T.E.; Chehboub, I.B.; Sotto, A.; Dunyach-Remy, C.; Lavigne, J.-P. Emergence of nasal carriage of ST80 and ST152 PVL+ *Staphylococcus aureus* isolates from livestock in Algeria. *Toxins* **2017**, *9*, 301.
10. Mairi, A.; Touati, A.; Pantel, A.; Zenati, K.; Martinez, A.Y.; Dunyach-Remy, C.; Sotto, A.; Lavigne, J.-P. Distribution of toxinogenic methicillin-resistant and methicillin-susceptible *Staphylococcus aureus* from different ecological niches in Algeria. *Toxins* **2019**, *11*, 500.
11. Ben Slama, K.; Gharsa, H.; Klibi, N.; Jouini, A.; Lozano, C.; Gómez-Sanz, E.; Zarazaga, M.; Boudabous, A.; Torres, C. Nasal carriage of *Staphylococcus aureus* in healthy humans with different levels of contact with animals in Tunisia: Genetic lineages, methicillin resistance, and virulence factors. *Eur. J. Clin. Microbiol. Infect. Dis.* **2011**, *30*, 499–508.
12. Ben Nejma, M.; Mastouri, M.; Bel Hadj Jrad, B.; Nour, M. Characterization of ST80 Panton-Valentine leukocidin-positive community-acquired methicillin-resistant *Staphylococcus aureus* clone in Tunisia. *Diagn. Microbiol. Infect. Dis.* **2013**, *77*, 20–24.
13. Ben, N.M.; Merghni, A.; Mastouri, M. Genotyping of Methicillin Resistant *Staphylococcus aureus* Strains Isolated from Hospitalized Children. *Int. J. Pediatr.* **2014**, *2014*, 314316.
14. Ahmed, M.O.; Baptiste, K.E.; Daw, M.A.; Elramalli, A.K.; Abouzeed, Y.M.; Petersen, A. Spa typing and identification of *pvl* genes of meticillin-resistant *Staphylococcus aureus* isolated from a Libyan hospital in Tripoli. *J. Glob. Antimicrob. Resist.* **2017**, *10*, 179–181.

15. Enany, S.; Yaoita, E.; Yoshida, Y.; Enany, M.; Yamamoto, T. Molecular characterization of Panton-Valentine leukocidin-positive community-acquired methicillin-resistant *Staphylococcus aureus* isolates in Egypt. *Microbiol. Res.* **2010**, *165*, 152–162.
16. Khalil, W.; Hashwa, F.; Shihabi, A.; Tokajian, S. Methicillin-resistant *Staphylococcus aureus* ST80-IV clone in children from Jordan. *Diagn. Microbiol. Infect. Dis.* **2012**, *73*, 228–230.
17. Al-Bakri, A.G.; Al-Hadithi, H.; Kasabri, V.; Othman, G.; Kriegeskorte, A.; Becker, K. The epidemiology and molecular characterization of methicillin-resistant *Staphylococci* sampled from a healthy Jordanian population. *Epidemiol. Infect.* **2013**, *141*, 2384–2391.
18. Bazzoun, D.A.; Harastani, H.H.; Shehabi, A.A.; Tokajian, S.T. Molecular typing of *Staphylococcus aureus* collected from a major hospital in Amman, Jordan. *J. Infect. Dev. Ctries* **2014**, *8*, 441–447.
19. Alkharsah, K.R.; Rehman, S.; Alkhamis, F.; Alnimr, A.; Diab, A.; Al-Ali, A.K. Comparative and molecular analysis of MRSA isolates from infection sites and carrier colonization sites. *Ann. Clin. Microbiol. Antimicrob.* **2018**, *17*, 7.
20. Monecke, S.; Skakni, L.; Hasan, R.; Ruppelt, A.; Ghazal, S.S.; Hakawi, A.; Slickers, P.; Ehricht, R. Characterisation of MRSA strains isolated from patients in a hospital in Riyadh, Kingdom of Saudi Arabia. *BMC Microbiol.* **2012**, *12*, 146.
21. Abou Shady, H.M.; Bakr, A.E.A.; Hashad, M.E.; Alzohairy, M.A. *Staphylococcus aureus* nasal carriage among outpatients attending primary health care centers: A comparative study of two cities in Saudi Arabia and Egypt. *Braz. J. Infect. Dis.* **2015**, *19*, 68–76.
22. El-Mahdy, T.S.; El-Ahmady, M.; Goering, R.V. Molecular characterization of methicillin-resistant *Staphylococcus aureus* isolated over a 2-year period in a Qatari hospital from multinational patients. *Clin. Microbiol. Infect.* **2014**, *20*, 169–173.
23. Udo, E.E.; O'Brien, F.G.; Al-Sweih, N.; Noronha, B.; Matthew, B.; Grubb, W.B. Genetic lineages of community-associated methicillin-resistant *Staphylococcus aureus* in Kuwait hospitals. *J. Clin. Microbiol.* **2008**, *46*, 3514–3516.
24. Udo, E.E.; Sarkhoo, E. The dissemination of ST80-SCC*mec*-IV community-associated methicillin resistant *Staphylococcus aureus* clone in Kuwait hospitals. *Ann. Clin. Microbiol. Antimicrob.* **2010**, *9*, 31.
25. Udo, E.E.; Sarkhoo, E. Genetic analysis of high-level mupirocin resistance in the ST80 clone of community-associated meticillin-resistant *Staphylococcus aureus*. *J. Med. Microbiol.* **2010**, *59*, 193–199.
26. Boswihi, S.S.; Udo, E.E.; Al-Sweih, N. Shifts in the clonal distribution of methicillin-resistant *Staphylococcus aureus* in Kuwait hospitals: 1992–2010. *PLoS ONE* **2016**, *11*, e0162744.
27. Alfouzan, W.; Dhar, R.; Udo, E. Genetic lineages of methicillin-resistant *Staphylococcus aureus* acquired during admission to an intensive care unit of a general hospital. *Med. Princ. Pract.* **2017**, *26*, 113–117.
28. Udo, E.E.; Al-Sweih, N. Dominance of community-associated methicillin-resistant *Staphylococcus aureus* clones in a maternity hospital. *PLoS ONE* **2017**, *12*, e0179563.
29. Tokajian, S.T.; Khalil, P.A.; Jabbour, D.; Rizk, M.; Farah, M.J.; Hashwa, F.A.; Araj, G.F. Molecular characterization of *Staphylococcus aureus* in Lebanon. *Epidemiol. Infect.* **2010**, *138*, 707–712.
30. Harastani, H.H.; Araj, G.F.; Tokajian, S.T. Molecular characteristics of *Staphylococcus aureus* isolated from a major hospital in Lebanon. *Int. J. Infect. Dis.* **2014**, *19*, 33–38.
31. Harastani, H.H.; Tokajian, S.T. Community-associated methicillin-resistant *Staphylococcus aureus* clonal complex 80 type IV (CC80-MRSA-IV) isolated from the Middle East: A heterogeneous expanding clonal lineage. *PLoS ONE* **2014**, *9*, e103715.
32. Ohadian Moghadam, S.; Modoodi Yaghooti, M.; Pourramezan, N.; Pourmand, M.R. Molecular characterization and antimicrobial susceptibility of the CA-MRSA isolated from healthcare workers, Tehran, Iran. *Microb. Pathog.* **2017**, *107*, 409–412.
33. Goudarzi, M.; Navidinia, M.; Beiranvand, E.; Goudarzi, H. Phenotypic and molecular characterization of methicillin-resistant *Staphylococcus aureus* clon*es carrying the Panton-Valentine leukocidin genes disseminating in Iranian hospitals. Microb. Drug Resist.* **2018**, *24*, 1543–1551.

34. Sonnevend, Á.; Blair, I.; Alkaabi, M.; Jumaa, P.; Al Haj, M.; Ghazawi, A.; Akawi, N.; Jouhar, F.S.; Hamadeh, M.B.; Pál, T. Change in meticillin-resistant *Staphylococcus aureus* clones at a tertiary care hospital in the United Arab Emirates over a 5-year period. *J. Clin. Pathol.* **2012**, *65*, 178–182.
35. Oksuz, L.; Dupieux, C.; Tristan, A.; Bes, M.; Etienne, J.; Gurler, N. The high diversity of MRSA clones detected in a university hospital in istanbul. *Int. J. Med. Sci.* **2013**, *10*, 1740–1745.
36. Udo, E.E.; Al-Lawati, B.A.-H.; Al-Muharmi, Z.; Thukral, S.S. Genotyping of methicillin-resistant *Staphylococcus aureus* in the Sultan Qaboos University Hospital, Oman reveals the dominance of Panton–Valentine leucocidin-negative ST6-IV/t304 clone. *New Microbes New Infect.* **2014**, *2*, 100–105.
37. Laham, N.A.; Mediavilla, J.R.; Chen, L.; Abdelateef, N.; Elamreen, F.A.; Ginocchio, C.C.; Pierard, D.; Becker, K.; Kreiswirth, B.N. MRSA clonal complex 22 strains harboring toxic shock syndrome toxin (TSST-1) are endemic in the primary hospital in Gaza, Palestine. *PLoS ONE* **2015**, *10*, e0120008.
38. Aung, K.T.; Hsu, L.Y.; Koh, T.H.; Hapuarachchi, H.C.; Chau, M.L.; Gutiérrez, R.A.; Ng, L.C. Prevalence of methicillin-resistant *Staphylococcus aureus* (MRSA) in retail food in Singapore. *Antimicrob. Resist. Infect. Control* **2017**, *6*, 94.
39. Haque, N.; Aung, M.S.; Paul, S.K.; Bari, M.S.; Ahmed, S.; Sarkar, S.R.; Roy, S.; Nasreen, S.A.; Mahmud, M.C.; Hossain, M.A.; et al. Molecular epidemiological characterization of methicillin-susceptible and -resistant *Staphylococcus aureus* isolated from skin and soft tissue infections in Bangladesh. *Microb. Drug Resist.* **2019**, *25*, 241–250.
40. Islam, M.A.; Parveen, S.; Rahman, M.; Huq, M.; Nabi, A.; Khan, Z.U.M.; Ahmed, N.; Wagenaar, J.A. Occurrence and characterization of methicillin resistant *Staphylococcus aureus* in processed raw foods and ready-to-eat foods in an urban setting of a developing country. *Front. Microbiol.* **2019**, *10*, 503.
41. Lim, K.T.; Hanifah, Y.A.; Yusof, M.Y.M.; Goering, R.V.; Thong, K.L. Temporal changes in the genotypes of methicillin-resistant *Staphylococcus aureus* strains isolated from a tertiary Malaysian hospital based on MLST, *spa*, and *mec*-associated *dru* typing. *Diagn. Microbiol. Infect. Dis.* **2012**, *74*, 106–112.
42. Aires de Sousa, M.; Bartzavali, C.; Spiliopoulou, I.; Sanches, I.S.; Crisóstomo, M.I.; de Lencastre, H. Two international methicillin-resistant *Staphylococcus aureus* clones endemic in a university hospital in Patras, Greece. *J. Clin. Microbiol.* **2003**, *41*, 2027–2032.
43. Vourli, S.; Perimeni, D.; Makri, A.; Polemis, M.; Voyiatzi, A.; Vatopoulos, A. Community acquired MRSA infections in a paediatric population in Greece. *Euro.Surveill.* **2005**, *10*, 78–79.
44. Chini, V.; Petinaki, E.; Meugnier, H.; Foka, A.; Bes, M.; Etienne, J.; Dimitracopoulos, G.; Spiliopoulou, I. Emergence of a new clone carrying Panton-Valentine leukocidin genes and staphylococcal cassette chromosome *mec* type V among methicillin-resistant *Staphylococcus aureus* in Greece. *Scand. J. Infect. Dis.* **2008**, *40*, 368–372.
45. Vourli, S.; Vagiakou, H.; Ganteris, G.; Orfanidou, M.; Polemis, M.; Vatopoulos, A.; Malamou-Ladas, H. High rates of community-acquired, Panton-Valentine leukocidin (PVL)-positive methicillin-resistant *S. aureus* (MRSA) infections in adult outpatients in Greece. Euro Surveill. **2009**, *14*, pii=19089.
46. Katopodis, G.D.; Grivea, I.N.; Tsantsaridou, A.J.; Pournaras, S.; Petinaki, E.; Syrogiannopoulos, G.A. Fusidic acid and clindamycin resistance in community-associated, methicillin-resistant *Staphylococcus aureus* infections in children of Central Greece. *BMC Infect. Dis.* **2010**, *10*, 351.
47. Hadjihannas, L.; Psichogiou, M.; Empel, J.; Kosmidis, C.; Goukos, D.; Bouzala, J.; Georgopoulos, S.; Malhotra-Kumar, S.; Harbarth, S.; Daikos, G.L.; et al. Molecular characteristics of community-associated methicillin-resistant *Staphylococcus aureus* colonizing surgical patients in Greece. *Diagn. Microbiol. Infect. Dis.* **2012**, *74*, 420–422.
48. Tsironi, E.E.; Zacharaki, F.; Grivea, I.N.; Tachmitzi, S.V.; Michoula, A.N.; Vlychou, M.; Petinaki, E.; Syrogiannopoulos, G.A. European ST80 community-associated methicillin-resistant *Staphylococcus aureus* orbital cellulitis in a neonate. *BMC Ophthalmol.* **2012**, *12*, 7.
49. Drougka, E.; Foka, A.; Liakopoulos, A.; Doudoulakakis, A.; Jelastopulu, E.; Chini, V.; Spiliopoulou, A.; Levidiotou, S.; Panagea, T.; Vogiatzi, A.; et al. A 12-year survey of methicillin-resistant *Staphylococcus aureus* infections in Greece: ST80-IV epidemic? *Clin. Microbiol. Infect.* **2014**, *20*, O796–O803.
50. Doudoulakakis, A.G.; Bouras, D.; Drougka, E.; Kazantzi, M.; Michos, A.; Charisiadou, A.; Spiliopoulou, I.; Lebessi, E.; Tsolia, M. Community-associated *Staphylococcus aureus* pneumonia among Greek children: Epidemiology, molecular characteristics, treatment, and outcome. *Eur. J. Clin. Microbiol. Infect. Dis.* **2016**, *35*, 1177–1185.

51. Drougka, E.; Foka, A.; Koutinas, C.K.; Jelastopulu, E.; Giormezis, N.; Farmaki, O.; Sarrou, S.; Anastassiou, E.D.; Petinaki, E.; Spiliopoulou, I. Interspecies spread of *Staphylococcus aureus* clones among companion animals and human close contacts in a veterinary teaching hospital. A cross-sectional study in Greece. *Prev. Vet. Med.* **2016**, *126*, 190–198.
52. Papadimitriou-Olivgeris, M.; Drougka, E.; Fligou, F.; Dodou, V.; Kolonitsiou, F.; Filos, K.S.; Anastassiou, E.D.; Petinaki, E.; Marangos, M.; Spiliopoulou, I. Spread of *tst*–positive *Staphylococcus aureus* strains belonging to ST30 clone among patients and healthcare workers in two intensive care units. *Toxins* **2017**, *9*, 270.
53. Nikolaras, G.P.; Papaparaskevas, J.; Samarkos, M.; Tzouvelekis, L.S.; Psychogiou, M.; Pavlopoulou, I.; Goukos, D.; Polonyfi, K.; Pantazatou, A.; Deliolanis, I.; et al. Changes in the Rates and Population Structure of MRSA from bloodstream infections. A Single Center Experience (2000–2015). *J. Glob. Antimicrob. Resist.* **2019**, *17*, 117–122.
54. Fang, H.; Hedin, G.; Li, G.; Nord, C.E. Genetic diversity of community-associated methicillin-resistant *Staphylococcus aureus* in southern Stockholm, 2000–2005. *Clin. Microbiol. Infect.* **2008**, *14*, 370–376.
55. Petersson, A.C.; Olsson-Liljequist, B.; Miörner, H.; Haeggman, S. Evaluating the usefulness of *spa* typing, in comparison with pulsed-field gel electrophoresis, for epidemiological typing of methicillin-resistant *Staphylococcus aureus* in a low-prevalence region in Sweden 2000–2004. *Clin. Microbiol. Infect.* **2010**, *16*, 456–462.
56. Urth, T.; Juul, G.; Skov, R.; Schønheyder, H.C. Spread of a methicillin-resistant *Staphylococcus aureus* ST80-IV clone in a Danish community. *Infect. Control Hosp. Epidemiol.* **2005**, *26*, 144–149.
57. Faria, N.A.; Oliveira, D.C.; Westh, H.; Monnet, D.L.; Larsen, A.R.; Skov, R.; de Lencastre, H. Epidemiology of emerging methicillin-resistant *Staphylococcus aureus* (MRSA) in Denmark: A nationwide study in a country with low prevalence of MRSA infection. *J. Clin. Microbiol.* **2005**, *43*, 1836–1842.
58. Bartels, M.D.; Boye, K.; Rhod Larsen, A.; Skov, R.; Westh, H. Rapid increase of genetically diverse methicillin-resistant *Staphylococcus aureus*, Copenhagen, Denmark. *Emerg. Infect. Dis.* **2007**, *13*, 1533–1540.
59. Larsen, A.R.; Stegger, M.; Böcher, S.; Sørum, M.; Monnet, D.L.; Skov, R.L. Emergence and characterization of community-associated methicillin-resistant *Staphyloccocus aureus* infections in Denmark, 1999 to 2006. *J. Clin. Microbiol.* **2009**, *47*, 73–78.
60. McLaws, F.B.; Larsen, A.R.; Skov, R.L.; Chopra, I.; O'Neill, A.J. Distribution of fusidic acid resistance determinants in methicillin-resistant *Staphylococcus aureus*. *Antimicrob. Agents Chemother.* **2011**, *55*, 1173–1176.
61. Bartels, M.D.; Larner-Svensson, H.; Meiniche, H.; Kristoffersen, K.; Schonning, K.; Nielsen, J.B.; Rohde, S.M.; Christensen, L.B.; Skibsted, A.W.; Jarlov, J.O.; et al. Monitoring meticillin resistant *Staphylococcus aureus* and its spread in Copenhagen, Denmark, 2013, through routine whole genome sequencing. *Euro Surveill.* **2015**, *20*, 20–28.
62. Edslev, S.M.; Westh, H.; Andersen, P.S.; Skov, R.; Kobayashi, N.; Bartels, M.D.; Vandenesch, F.; Petersen, A.; Worning, P.; Larsen, A.R.; et al. Identification of a PVL-negative SCC*mec*-IVa sublineage of the methicillin-resistant *Staphylococcus aureus* CC80 lineage: Understanding the clonal origin of CA-MRSA. *Clin. Microbiol. Infect.* **2018**, *24*, 273–278.
63. Goering, R.V.; Larsen, A.R.; Skov, R.; Tenover, F.C.; Anderson, K.L.; Dunman, P.M. Comparative genomic analysis of European and Middle Eastern community-associated methicillin-resistant *Staphylococcus aureus* (CC80:ST80-IV) isolates by high-density microarray. *Clin. Microbiol. Infect.* **2009**, *15*, 748–755.
64. Hanssen, A.-M.; Fossum, A.; Mikalsen, J.; Halvorsen, D.S.; Bukholm, G.; Sollid, J.U.E. Dissemination of community-acquired methicillin-resistant *Staphylococcus aureus* clones in northern Norway: Sequence types 8 and 80 predominate. *J. Clin. Microbiol.* **2005**, *43*, 2118–2124.
65. Fossum, A.E.; Bukholm, G. Increased incidence of methicillin-resistant *Staphylococcus aureus* ST80, novel ST125 and SCC*mec*IV in the south-eastern part of Norway during a 12-year period. *Clin. Microbiol. Infect.* **2006**, *12*, 627–633.
66. Monecke, S.; Aamot, H.V.; Stieber, B.; Ruppelt, A.; Ehricht, R. Characterization of PVL-positive MRSA from Norway. *APMIS* **2014**, *122*, 580–584.
67. Dauwalder, O.; Lina, G.; Durand, G.; Bes, M.; Meugnier, H.; Jarlier, V.; Coignard, B.; Vandenesch, F.; Etienne, J.; Laurent, F. Epidemiology of invasive methicillin-resistant *Staphylococcus aureus* clones collected in France in 2006 and 2007. *J. Clin. Microbiol.* **2008**, *46*, 3454–3458.

68. Maugat, S.; de Rougemont, A.; Aubry-Damon, H.; Reverdy, M.-E.; Georges, S.; Vandenesch, F.; Etienne, J.; Coignard, B. Methicillin-resistant *Staphylococcus aureus* among a network of French private-sector community-based-medical laboratories. *Med. Mal. Infect.* **2009**, *39*, 311–318.
69. Del Giudice, P.; Bes, M.; Hubiche, T.; Blanc, V.; Roudière, L.; Lina, G.; Vandenesch, F.; Etienne, J. Panton-Valentine leukocidin-positive *Staphylococcus aureus* strains are associated with follicular skin infections. *Dermatology* **2011**, *222*, 167–170.
70. Robert, J.; Tristan, A.; Cavalié, L.; Decousser, J.-W.; Bes, M.; Etienne, J.; Laurent, F.; ONERBA (Observatoire National de l'Epidémiologie de Résistance Bactérienne aux Antibiotiques). Panton-valentine leukocidin-positive and toxic shock syndrome toxin 1-positive methicillin-resistant *Staphylococcus aureus*: A French multicenter prospective study in 2008. *Antimicrob. Agents Chemother.* **2011**, *55*, 1734–1739.
71. Lamy, B.; Laurent, F.; Gallon, O.; Doucet-Populaire, F.; Etienne, J.; Decousser, J.-W.; Collège de Bactériologie Virologie Hygiène (ColBVH) Study Group. Antibacterial resistance, genes encoding toxins and genetic background among *Staphylococcus aureus* isolated from community-acquired skin and soft tissue infections in France: A national prospective survey. *Eur. J. Clin. Microbiol. Infect. Dis.* **2012**, *31*, 1279–1284.
72. van der Mee-Marquet, N.; Poisson, D.-M.; Lavigne, J.-P.; Francia, T.; Tristan, A.; Vandenesch, F.; Quentin, R.; Bertrand, X. The incidence of *Staphylococcus aureus* ST8-USA300 among French pediatric inpatients is rising. *Eur. J. Clin. Microbiol. Infect. Dis.* **2015**, *34*, 935–942.
73. Monecke, S.; Slickers, P.; Hotzel, H.; Richter-Huhn, G.; Pohle, M.; Weber, S.; Witte, W.; Ehricht, R. Microarray-based characterisation of a Panton-Valentine leukocidin-positive community-acquired strain of methicillin-resistant *Staphylococcus aureus*. *Clin. Microbiol. Infect.* **2006**, *12*, 718–728.
74. Monecke, S.; Slickers, P.; Ellington, M.J.; Kearns, A.M.; Ehricht, R. High diversity of Panton-Valentine leukocidin-positive, methicillin-susceptible isolates of *Staphylococcus aureus* and implications for the evolution of community-associated methicillin-resistant *S. aureus*. *Clin. Microbiol. Infect.* **2007**, *13*, 1157–1164.
75. Witte, W.; Pasemann, B.; Cuny, C. Detection of low-level oxacillin resistance in *mecA*-positive *Staphylococcus aureus*. *Clin. Microbiol. Infect.* **2007**, *13*, 408–412.
76. Witte, W.; Strommenger, B.; Cuny, C.; Heuck, D.; Nuebel, U. Methicillin-resistant *Staphylococcus aureus* containing the Panton-Valentine leucocidin gene in Germany in 2005 and 2006. *J. Antimicrob. Chemother.* **2007**, *60*, 1258–1263.
77. Monecke, S.; Jatzwauk, L.; Weber, S.; Slickers, P.; Ehricht, R. DNA microarray-based genotyping of methicillin-resistant *Staphylococcus aureus* strains from Eastern Saxony. *Clin. Microbiol. Infect.* **2008**, *14*, 534–545.
78. Schaumburg, F.; Köck, R.; Mellmann, A.; Richter, L.; Hasenberg, F.; Kriegeskorte, A.; Friedrich, A.W.; Gatermann, S.; Peters, G.; von Eiff, C.; et al. Population Dynamics among Methicillin-Resistant *Staphylococcus aureus* Isolates in Germany during a 6-Year Period. *J. Clin. Microbiol.* **2012**, *50*, 3186–3192.
79. Klein, S.; Menz, M.-D.; Zanger, P.; Heeg, K.; Nurjadi, D. Increase in the prevalence of Panton-Valentine leukocidin and clonal shift in community-onset methicillin-resistant *Staphylococcus aureus* causing skin and soft-tissue infections in the Rhine-Neckar Region, Germany, 2012–2016. *Int. J. Antimicrob. Agents* **2019**, *53*, 261–267.
80. Holmes, A.; Ganner, M.; McGuane, S.; Pitt, T.L.; Cookson, B.D.; Kearns, A.M. *Staphylococcus aureus* isolates carrying Panton-Valentine leucocidin genes in England and Wales: Frequency, characterization, and association with clinical disease. *J. Clin. Microbiol.* **2005**, *43*, 2384–2390.
81. Otter, J.A.; French, G.L. The emergence of community-associated methicillin-resistant *Staphylococcus aureus* at a London teaching hospital, 2000–2006. *Clin. Microbiol. Infect.* **2008**, *14*, 670–676.
82. Denis, O.; Deplano, A.; De Beenhouwer, H.; Hallin, M.; Huysmans, G.; Garrino, M.G.; Glupczynski, Y.; Malaviolle, X.; Vergison, A.; Struelens, M.J. Polyclonal emergence and importation of community-acquired methicillin-resistant *Staphylococcus aureus* strains harbouring Panton-Valentine leucocidin genes in Belgium. *J. Antimicrob. Chemother.* **2005**, *56*, 1103–1106.
83. Brauner, J.; Hallin, M.; Deplano, A.; De Mendonça, R.; Nonhoff, C.; De Ryck, R.; Roisin, S.; Struelens, M.J.; Denis, O. Community-acquired methicillin-resistant *Staphylococcus aureus* clones circulating in Belgium from 2005 to 2009: Changing epidemiology. *Eur. J. Clin. Microbiol. Infect. Dis.* **2013**, *32*, 613–620.

84. Peeters, L.E.J.; Argudín, M.A.; Azadikhah, S.; Butaye, P. Antimicrobial resistance and population structure of *Staphylococcus aureus* recovered from pigs farms. *Vet. Microbiol.* **2015**, *180*, 151–156.
85. Cercenado, E.; Cuevas, O.; Marín, M.; Bouza, E.; Trincado, P.; Boquete, T.; Padilla, B.; Vindel, A. Community-acquired methicillin-resistant *Staphylococcus aureus* in Madrid, Spain: Transcontinental importation and polyclonal emergence of Panton-Valentine leukocidin-positive isolates. *Diagn. Microbiol. Infect. Dis.* **2008**, *61*, 143–149.
86. Vindel, A.; Trincado, P.; Cuevas, O.; Ballesteros, C.; Bouza, E.; Cercenado, E. Molecular epidemiology of community-associated methicillin-resistant *Staphylococcus aureus* in Spain: 2004–2012. *J. Antimicrob. Chemother.* **2014**, *69*, 2913–2919.
87. Sanchini, A.; Campanile, F.; Monaco, M.; Cafiso, V.; Rasigade, J.-P.; Laurent, F.; Etienne, J.; Stefani, S.; Pantosti, A. DNA microarray-based characterisation of Panton-Valentine leukocidin-positive community-acquired methicillin-resistant *Staphylococcus aureus* from Italy. *Eur. J. Clin. Microbiol. Infect. Dis.* **2011**, *30*, 1399–1408.
88. Aschbacher, R.; Pichon, B.; Spoladore, G.; Pagani, E.; Innocenti, P.; Moroder, L.; Ganner, M.; Hill, R.; Pike, R.; Ganthaler, O.; et al. High clonal heterogeneity of Panton-Valentine leukocidin-positive meticillin-resistant *Staphylococcus aureus* strains from skin and soft-tissue infections in the Province of Bolzano, Northern Italy. *Int. J. Antimicrob. Agents* **2012**, *39*, 522–525.
89. Rossney, A.S.; Shore, A.C.; Morgan, P.M.; Fitzgibbon, M.M.; O'Connell, B.; Coleman, D.C. The emergence and importation of diverse genotypes of methicillin-resistant *Staphylococcus aureus* (MRSA) harboring the Panton-Valentine leukocidin gene (*pvl*) reveal that *pvl* is a poor marker for community-acquired MRSA strains in Ireland. *J. Clin. Microbiol.* **2007**, *45*, 2554–2563.
90. Shore, A.C.; Tecklenborg, S.C.; Brennan, G.I.; Ehricht, R.; Monecke, S.; Coleman, D.C. Panton-Valentine leukocidin-positive *Staphylococcus aureus* in Ireland from 2002 to 2011: 21 clones, frequent importation of clones, temporal shifts of predominant methicillin-resistant *S. aureus* clones, and increasing multiresistance. *J. Clin. Microbiol.* **2014**, *52*, 859–870.
91. Wannet, W.J.B.; Spalburg, E.; Heck, M.E.O.C.; Pluister, G.N.; Tiemersma, E.; Willems, R.J.L.; Huijsdens, X.W.; de Neeling, A.J.; Etienne, J. Emergence of virulent methicillin-resistant *Staphylococcus aureus* Strains carrying Panton-Valentine leucocidin genes in The Netherlands. *J. Clin. Microbiol.* **2005**, *43*, 3341–3345.
92. Huijsdens, X.W.; van Lier, A.M.C.; van Kregten, E.; Verhoef, L.; van Santen-Verheuvel, M.G.; Spalburg, E.; Wannet, W.J.B. Methicillin-resistant *Staphylococcus aureus* in Dutch soccer team. *Emerg. Infect. Dis.* **2006**, *12*, 1584–1586.
93. Stam-Bolink, E.M.; Mithoe, D.; Baas, W.H.; Arends, J.P.; Möller, A.V.M. Spread of a methicillin-resistant *Staphylococcus aureus* ST80 strain in the community of the northern Netherlands. *Eur. J. Clin. Microbiol. Infect. Dis.* **2007**, *26*, 723–727.
94. Nulens, E.; Stobberingh, E.E.; Smeets, E.; van Dessel, H.; Welling, M.A.; Sebastian, S.; van Tiel, F.H.; Beisser, P.S.; Deurenberg, R.H. Genetic diversity of methicillin-resistant *Staphylococcus aureus* in a tertiary hospital in The Netherlands between 2002 and 2006. *Eur. J. Clin. Microbiol. Infect. Dis.* **2009**, *28*, 631–639.
95. Hetem, D.J.; Westh, H.; Boye, K.; Jarløv, J.O.; Bonten, M.J.M.; Bootsma, M.C.J. Nosocomial transmission of community-associated methicillin-resistant *Staphylococcus aureus* in Danish Hospitals. *J. Antimicrob. Chemother.* **2012**, *67*, 1775–1780.
96. Krziwanek, K.; Luger, C.; Sammer, B.; Stumvoll, S.; Stammler, M.; Metz-Gercek, S.; Mittermayer, H. PVL-positive MRSA in Austria. *Eur. J. Clin. Microbiol. Infect. Dis.* **2007**, *26*, 931–935.
97. Berktold, M.; Grif, K.; Mäser, M.; Witte, W.; Würzner, R.; Orth-Höller, D. Genetic characterization of Panton-Valentine leukocidin-producing methicillin-resistant *Staphylococcus aureus* in Western Austria. *Wien. Klin. Wochenschr.* **2012**, *124*, 709–715.
98. Fenner, L.; Widmer, A.F.; Dangel, M.; Frei, R. Distribution of *spa* types among meticillin-resistant *Staphylococcus aureus* isolates during a 6 year period at a low-prevalence University Hospital. *J Med Microbiol* **2008**, *57*, 612–616.

99. Budimir, A.; Deurenberg, R.H.; Bosnjak, Z.; Stobberingh, E.E.; Cetkovic, H.; Kalenic, S. A variant of the Southern German clone of methicillin-resistant *Staphylococcus aureus* is predominant in Croatia. *Clin. Microbiol. Infect.* **2010**, *16*, 1077–1083.
100. Scicluna, E.A.; Shore, A.C.; Thürmer, A.; Ehricht, R.; Slickers, P.; Borg, M.A.; Coleman, D.C.; Monecke, S. Characterisation of MRSA from Malta and the description of a Maltese epidemic MRSA strain. *Eur. J. Clin. Microbiol. Infect. Dis.* **2010**, *29*, 163–170.
101. Conceição, T.; Aires-de-Sousa, M.; Pona, N.; Brito, M.J.; Barradas, C.; Coelho, R.; Sardinha, T.; Sancho, L.; de Sousa, G.; do Machado, M.C.; et al. High prevalence of ST121 in community-associated methicillin-susceptible *Staphylococcus aureus* lineages responsible for skin and soft tissue infections in Portuguese children. *Eur. J. Clin. Microbiol. Infect. Dis.* **2011**, *30*, 293–297.
102. Dermota, U.; Jurca, T.; Harlander, T.; Košir, M.; Zajc, U.; Golob, M.; Zdovc, I.; Košnik, I.G. Infections caused by community-associated methicillin-resistant *Staphylococcus aureus* european clone (ST80) in Slovenia between 2006 and 2013. *Zdr. Varst* **2016**, *55*, 121–125.
103. Fluit, A.C.; Carpaij, N.; Majoor, E.A.M.; Weinstein, R.A.; Aroutcheva, A.; Rice, T.W.; Bonten, M.J.M.; Willems, R.J.L. Comparison of an ST80 MRSA strain from the USA with European ST80 strains. *J. Antimicrob. Chemother.* **2015**, *70*, 664–669.
